# Theoretical–experimental study of new synthesized hydrazine–hydrazone benzenesulfonamide inhibitors for carbon steel in a 1.0 M HCI DFT, QSAR

**DOI:** 10.1038/s41598-026-39947-w

**Published:** 2026-05-29

**Authors:** Walid E. Elgammal, Reema H. Aldahiri, Shereen M. Al-Shomar, Saber M. Hassan, Amr Gangan, N. S. Abdelshafi

**Affiliations:** 1 Chemistry Department, Faculty of Science (Boys), Al-Azhar University, Nasr City, 11884 Cairo Egypt; 2 Department of Chemistry, College of Science, University of Jeddah, Jeddah, 21959 Saudi Arabia; 3 Department of Physics, College of Science, University of Ha’il, P.O. Box 2440, Ha’il, Saudi Arabia; 4 Chemistry Department, Faculty of Education, Ain Shams University, Roxy, 11711 Cairo Egypt; 5 Chemistry Department, Faculty of Science, Al-Azhar University, Nasr City, Cairo, Egypt; 6 Al-Azhar University, El-Nasr Road, Nasr City, 11884 Cairo Egypt

**Keywords:** Hydrazone inhibitors, Carbon steel, AC and DC techniques, Anticorrosion, QSAR, DFT, Chemistry, Materials science

## Abstract

In this study, quantitative structure-activity relationship (QSAR) modeling using genetic function approximation was applied to predict the corrosion inhibition efficiency of three synthesized hydrazone derivatives, IHE, BHE, and AHE, on N80 carbon steel in 1.0 M HCl solution at various concentrations (50–400 ppm) and temperatures (298–358 K). The inhibitors’ performance was evaluated via electrochemical impedance spectroscopy (EIS), potentiodynamic polarization (PDP), weight loss (WL), and surface analysis techniques. Corrosion inhibition increased with concentration, reaching maximum efficiencies of 90.34% (IHE), 85.93% (BHE), and 67.97% (AHE) at 400 ppm. Adsorption followed the Langmuir isotherm, indicating monolayer formation. High adsorption equilibrium constants (K_ads_) and negative free energy changes ($\:{{\Delta\:}\mathrm{G}}_{\mathrm{a}\mathrm{d}\mathrm{s}}^{\mathrm{o}}$: − 36.00, − 32.78, − 30.05 kJ/mol) confirmed strong, spontaneous adsorption of the inhibitors onto the steel surface. Density functional theory (DFT) and molecular simulations further revealed favorable electronic properties and stable adsorption behavior, supporting the experimental results. The combined theoretical and experimental data highlight the efficiency of the hydrazone derivatives as promising corrosion inhibitors for carbon steel in acidic environments.

**Supplementary Information:**

The online version contains supplementary material available at 10.1038/s41598-026-39947-w.

## Introduction

Corrosion represents the critical degradation process that is observed when metallic components in industrial systems are subjected to aggressive environments during service^1–5^. This phenomenon not only leads to the unnecessary depletion of natural resources but also results in substantial economic losses^6^. Consequently, the development of effective corrosion mitigation strategies has remained a subject of significant interest within the scientific and engineering communities^7,8^. Various industries extensively employ N80CS, an alloy primarily consisting of iron with carbon, due to its resilience, longevity, and affordability^9^. It is frequently used in beams, columns, machinery, automotive components, pipes, tanks, and pressure vessels for oil, gas, and water transportation.^10^. On the other hand, although carbon steel is widely utilized, it has a tendency to undergo corrosion, particularly in stressful environments that contain moisture, salts, acids, or gases like CO₂ and H₂S. Metallic corrosion in carbon steel structures can cause considerable material degradation, jeopardizing structural integrity and resulting in economic losses^11^. It is known that corrosion inhibitors are chemicals introduced into environments or materials to reduce the pace or halt the process of corrosion. They operate by either creating a safeguard coating on the N80CS apparent or through neutralizing corrosive agents in the environment. Such compounds frequently serve as mixed inhibitors, targeting anodic corrosion, metal atoms losing electrons, in addition to cathodic corrosion, the gain of electrons by other substances, and creating a shielding water-repellent layer on the metallic surface. Heteroatoms (S, N, and O) incorporation with efficient sets (OH, NH₂, COOH, etc.)^12,14^ facilitate the adsorption process of inhibitors. Organic derivative inhibitors serve as a physical barrier to chlorides in acidic corrosive solutions and provide an electrostatic revulsion through -Ve charge terminal groups^14,15^. Hydrazone compounds are a unique subgroup of Schiff bases recognized for forming crystals, which makes them easier to purify. They are typically synthesized through one of three approaches: via condensation reactions between aldehydes or ketones and hydrazines; by the reaction of Japp-Klingemann, where aryldiazonium salts react with beta-keto acids or esters; or by coupling aryl halides with substituted hydrazones. The general formula for hydrazones is R_1_R_2_C = N-NH. These compounds contain two nitrogen atoms that, although both nucleophilic, have distinct reactivity. The amino nitrogen is particularly reactive, a trait enhanced by the interaction of the double bonds between carbon and nitrogen and an unshared electron pair on the terminal nitrogen atoms. Furthermore, carbons in hydrazone structures have a dual capacity to act as both an electrophile and a nucleophile^16–18^.

In the area of corrosion prevention, hydrazones have garnered considerable interest for their anti-corrosive properties. These compounds have proven to be effective employed as substances that prevent corrosion for metals such as copper, nickel, and steel in both acidic and alkaline conditions^19–22^. Conversely, the computational assessment of inhibitors adsorption mechanisms has become crucial for a better understanding of how organic compounds inhibit corrosion. QSAR is a vital technique in computational chemistry that concentrates on creating mathematical relationships between the chemical structures of compounds and their activities. This enables researchers to forecast the properties of new molecules based on existing data^23^.

Throughout this investigation, we study the preparation and application of novel hydrazone derivatives, **IHE**, **BHE**, and **AHE**. Their protective ability for carbon steel corrosion is assessed in acidic environments by employing EIS and PDP techniques. To examine the corrosion and inhibition effects on N80 carbon steel surfaces, we used SEM/EDX, contact angle, and AFM. Moreover, DFT and MD simulations were conducted to explore in greater detail the nature of the interactions between hydrazones and the Fe(110) surface.

## Resources and procedures

### Substances

Commercial vendors supplied the solvents and preparative reagents. A common technique for monitoring reaction progress and determining product purity was done. The carbon steel sample was sourced from the Central Metallurgical Research Institute in Egypt and fashioned into disc-shaped specimens with a 10 mm diameter. The carbon steel sample was analyzed by FOUNDRY-MASTER Optical Emission Spectrometer. The composition of it (in wt %) is as follows: 4.8% C, 2.7% O, and 92.5% Fe. (**Supplementary Methods**)

### Electrochemical and gravimetric weight loss measurements

Electrochemical experiments were measured by the AC impedance technique (EIS). These experiments utilized a galvanostat/potentiostat/ZRA analyzer (GAMRY Reference 3000) with a traditional configuration featuring a mercury/mercurous chloride (Hg/Hg₂Cl₂) reference electrode in a saturated KCl solution and platinum wire mesh serving as the secondary electrode. N80 carbon steel coupons (7 cm × 2 cm × 1 mm) were precisely weighed and then submerged in 100 mL of a 1.0 M hydrochloric acid (HCl) solution under conditions including and excluding a range of **AHE**, **BHE**, and **IHE** inhibitor concentrations. Corrosion tests were conducted over a 24-hour period at temperatures varying between 298 K and 358 K. (**Supplementary Methods**)

### N80 carbon steel surface examination

The confirmation of **AHE**,** BHE**,** and IHE** inhibitors adsorption was established through the surface characterization of N80CS specimens measuring$\:\:2\times\:2.50\times\:0.04\:\mathrm{c}\mathrm{m}$. This characterization was conducted both before the inhibition process and after via immersing the testers in 1.0 M corrosive solutions, both with besides without the existence of 400 ppm inhibitors, for a duration of one day at 298 K. The surfaces were analyzed by SEM, the scanning electron microscope, (EDX) characterization, energy dispersive X-ray analysis, and scanning probe technique using atomic-scale forces (AFM). Additionally, the angle of static contact measurements was taken. (**Supplementary Methods**)

### Theoretical analysis

QSAR, the module DMol^3^, and the module of Monte Carlo simulation (MC) are in the software, Material Studio 7.0, by BIOVIA. DMol^3^has been employed for calculations of quantum chemicals using DFT, the density functional theory approach, to assess global reactivates and local reactivates of the **AHE**,** BHE**,** and IHE** inhibitors^24,25^. (**Supplementary Methods**)

### Synthesis and characterization of predicted inhibitors

**Scheme 1:**
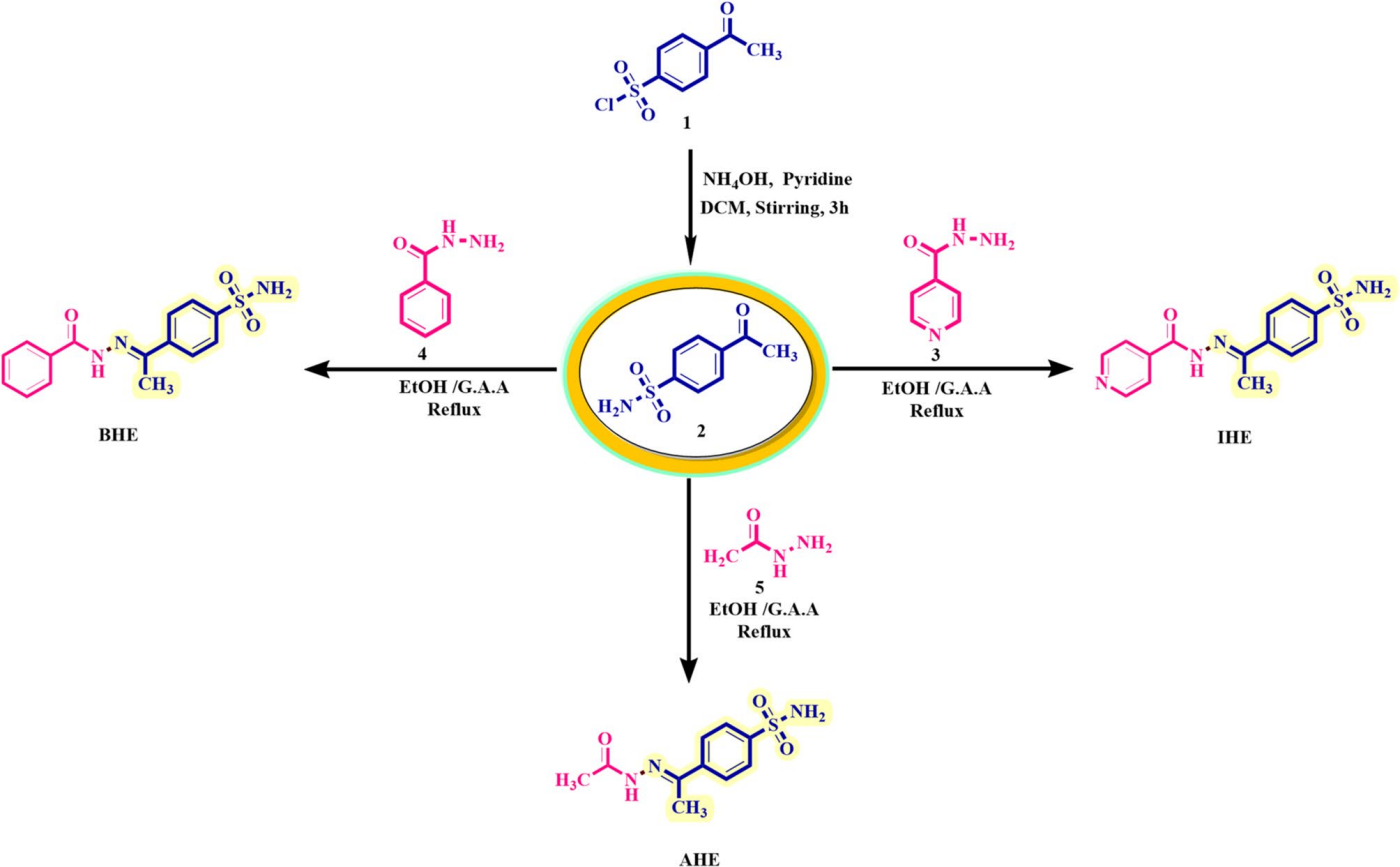
Synthetic procedure of sulfonamide–N-arylhydrazone hybrid.

A sequential process was utilized to synthesize innovative carbon steel inhibitors, as shown in Scheme 1. The process started with the reaction of 4-acetylbenzene sulfonyl chloride **1** ^26,27^ with an ammonia solution (25%) under basic-catalyzed at 23–25 °C to yield 4-acetylbenzenesulfonamide **2** ^28^. On the other hand, the treatment of substituted ketone 2 with electron-rich amino compounds like hydrazides **3–5** ^29^ and then the resulting mixture was subjected to heating in an acid-catalyzed alcoholic solvent. This process selectively adds the amino-hydrazide nucleophile to the ketone’s carbonyl group, resulting in the formation of the desired hydrazones (**IHE**,** BHE**,** and AHE**) (Scheme 1). (**Supplementary Methods**)

**Scheme 2:**
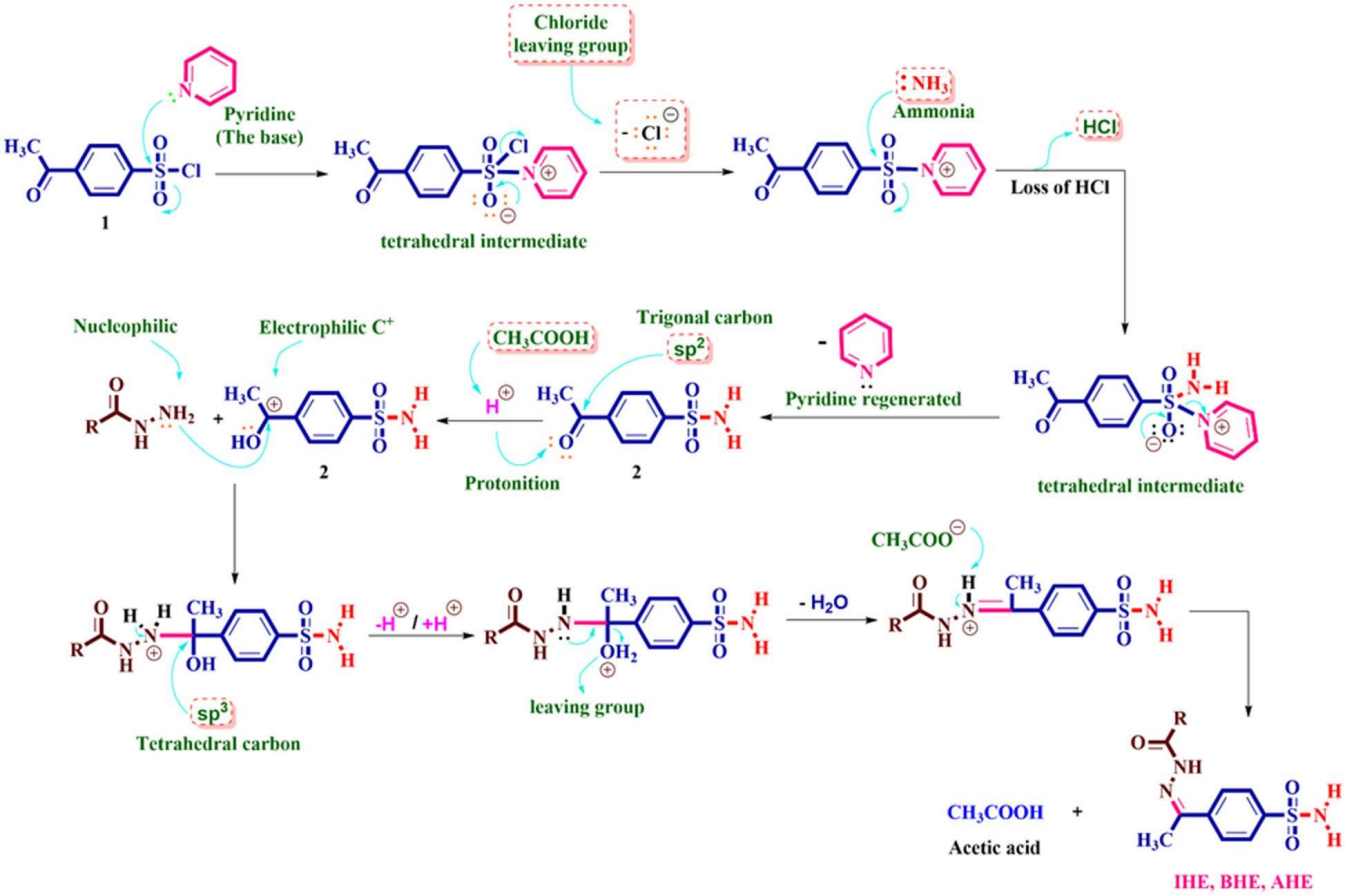
Suggest a reasonable mechanism for the establishment of sulfonamide 2 and N-arylhydrazones (IHE, BHE, and AHE).

The formation of the sulfonamide derivative **2** can be explained by referring to the plausible mechanism described in Scheme 2. In the initial step, the sulfonyl group is activated for further reaction with a nucleophile through its reaction with pyridine, which leads to the formation of adduct from the pyridinium salt via the elimination of chloride ion (-Cl^−^). In the second stage, the ammonium hydroxide (acts as nucleophilic) attacks the sulfur atom of the sulfonyl group (acts as electrophilic), this leads to the formation of a non-separate intermediate, which tolerates pyridine losses to produce the desired sulfonamide derivative **2**. Finally, the formation of N-arylhydrazones (**IHE**,** BHE**,** and AHE**) through the condensation reaction between benzohydrazide and the corresponding ketone **2**, which encompasses an acid-base interaction between the catalyst and the oxygen of the ketone carbonyl group (**sp**^**2**^, **> C = O** ) moiety, induces the generation of an electrophilic carbon atom (C^+^). Afterward, carbonyl functionality can undergo an intermolecular nucleophilic condensation of the amine moiety from the hydrazide to form the non-isolated intermediates, which may go through dehydration to afford desirable N-arylhydrazones (**IHE**,** BHE**,** and AHE**).

**Fig. 1:**
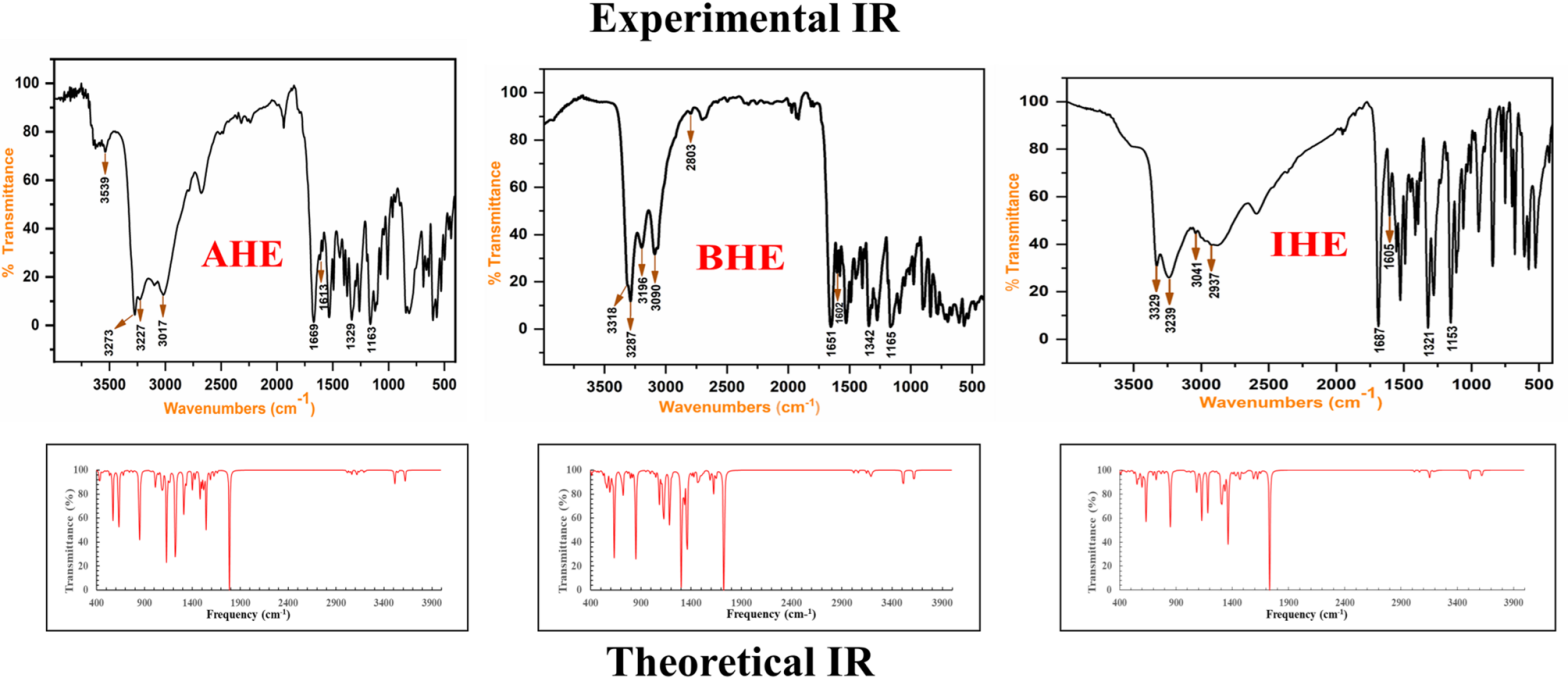
Experimental and theoretical FT-IR spectrum for **AHE**,** BHE**, and **IHE** inhibitors.

**Fig. 2:**
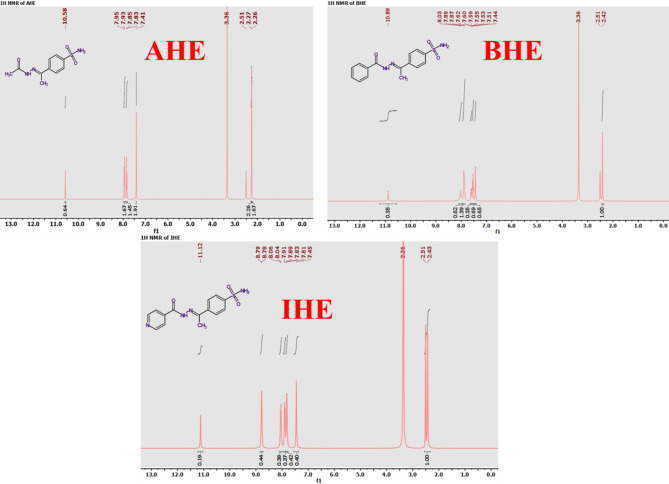
^1^H NMR spectrum for AHE, BHE, and IHE inhibitors.

**Fig. 3:**
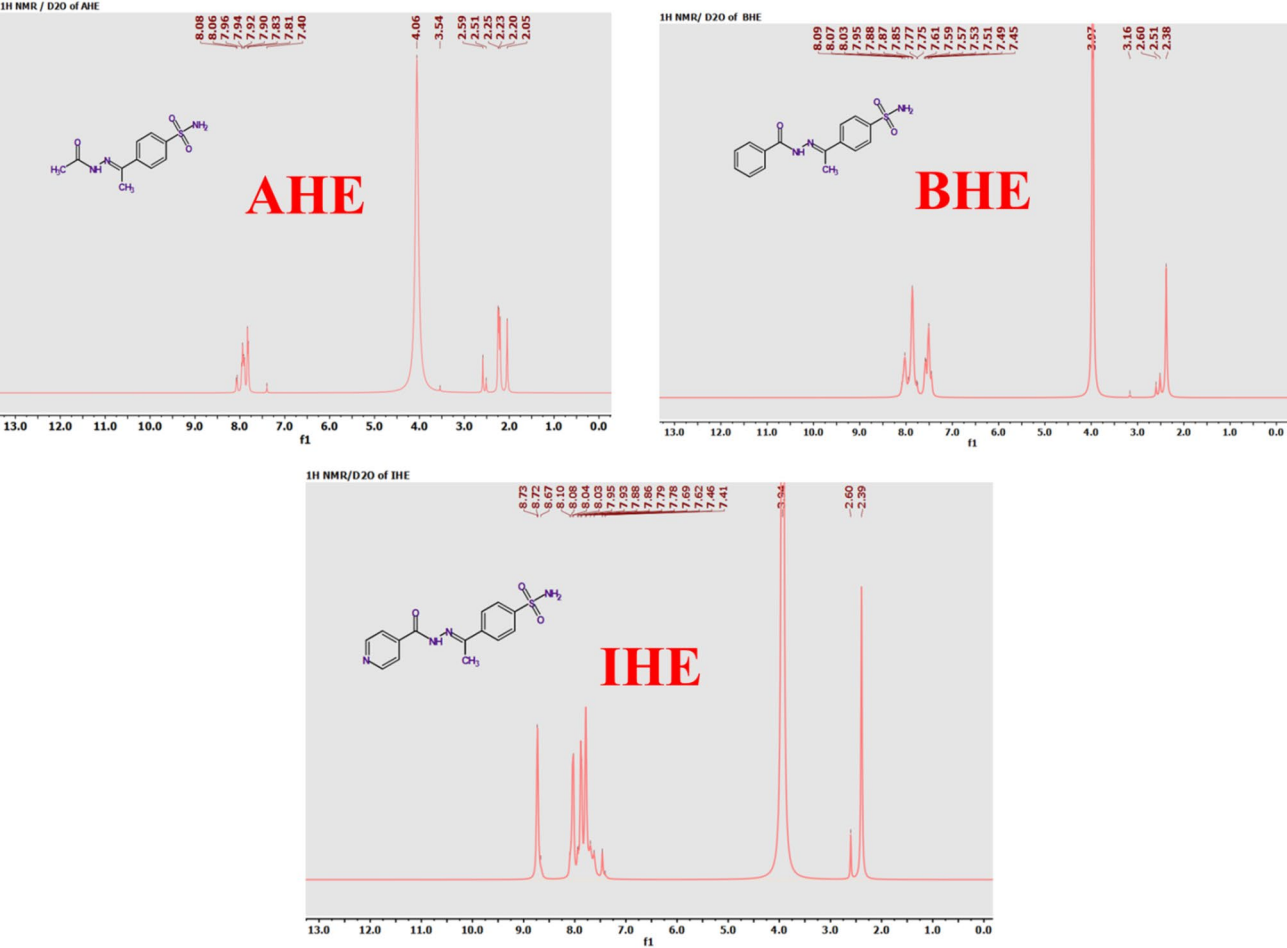
^1^H NMR/D_2_O spectrum for AHE, BHE, and IHE inhibitors.

**Fig. 4:**
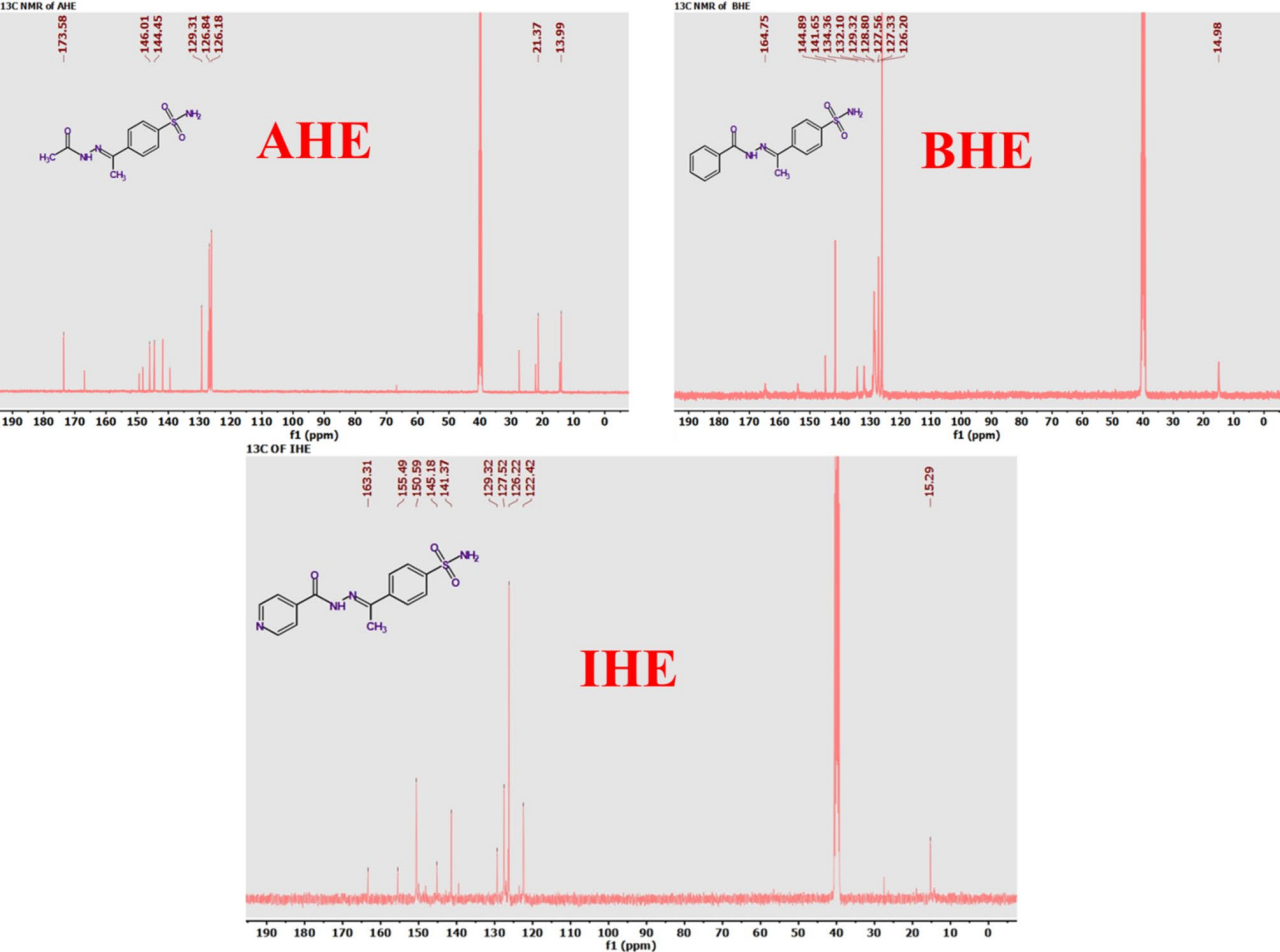
^13^C NMR spectrum for **AHE**,** BHE**, and **IHE** inhibitors.

**Fig. 5:**
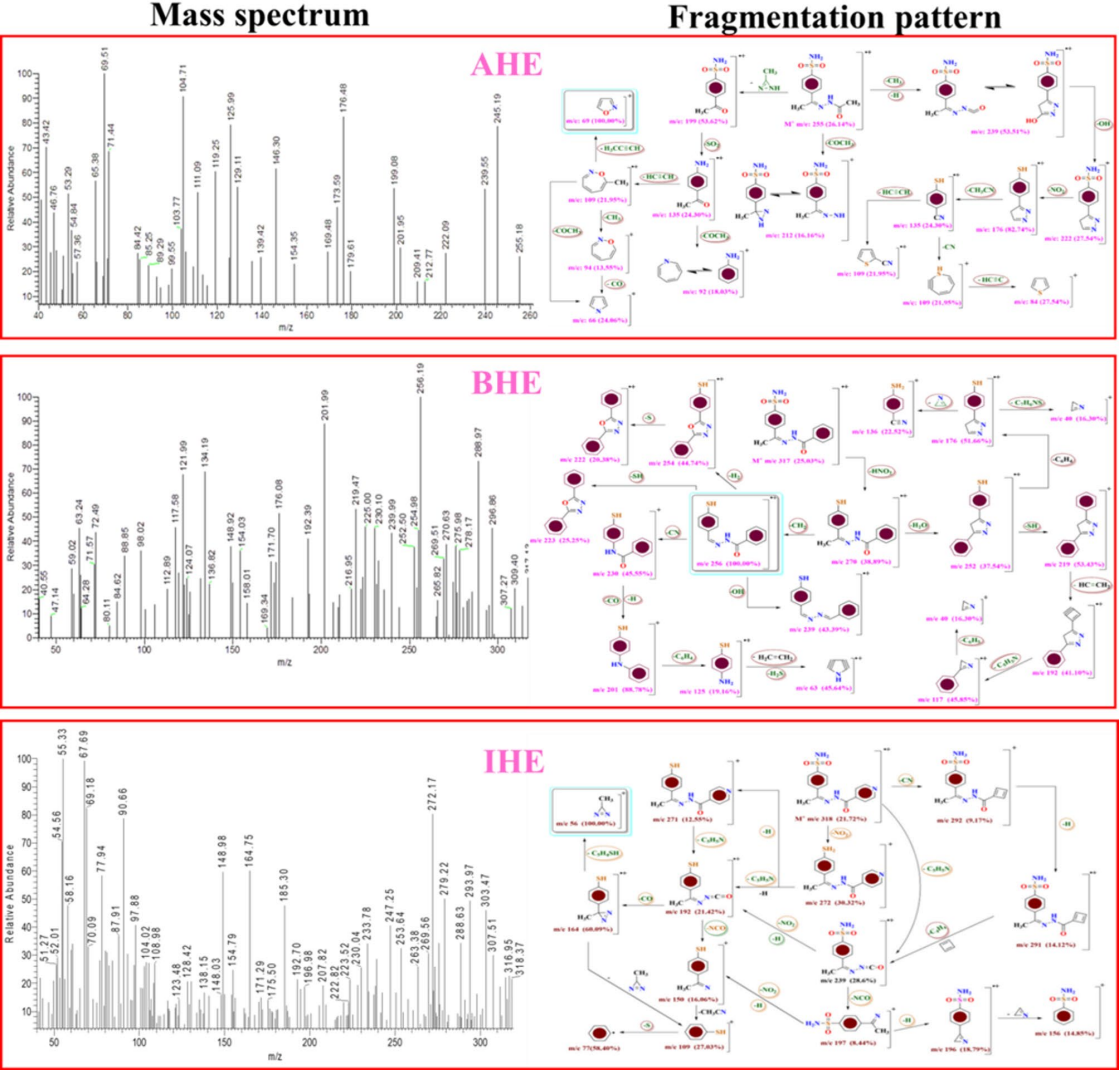
Mass spectrum and Fragmentation pattern of **AHE**,** BHE**, and **IHE** inhibitors by EI-MS.

The new corrosion inhibitors’ chemical makeup has been demonstrated using spectroscopic measurements, including ^1^H/^13^C nuclear magnetic resonance and FT-IR (Figs. 1, 2, 3 and 4), besides mass spectroscopy (Fig. 5). The Experimental and theoretical FT-IR spectrum revealed that the distinctive ketone carbonyl peak had vanished and that an absorption band had appeared in the 1688–1650 cm⁻¹ regions. This absorption band may have been brought on by the hydrazone group’s C = O stretching vibrations. Additionally, the extending vibrations of the imine group and amino group, respectively, were identified as the source of the peaks in the area of 3540 –3190 cm⁻¹. The proton nuclear magnetic resonance (proton NMR) confirms the being of two singlet signals linked to the hydrogens of the imine unit and the NH₂ unit. These signals are canceled by D_2_O. On the other hand, the carbon-13 NMR spectroscopy shows resonance in the range of 175 − 162 ppm, which belongs to the carbonyl carbon of the hydrazine moiety. It also exhibits distinct signals that support the proposed chemical structure for the prepared compounds, and also, the electron ionization mass spectroscopy (EI-MS) of the inhibitors records the original ion peak (mass-to-charge ratio) (m/z) [M]^+^ as 318.35, 317.36, and 255.29, apiece. These findings closely align with the measured values for their molecular formulas. (**Supplementary Methods)**

Comparative analyses between experimental and theoretically derived chemical shifts are systematically detailed in Table 1, with validation assessed through RMSD values. For inhibitor **AHE**, the anticipated concordance was observed, yielding RMSD values of 1.30 for 1 H NMR and 5.06 for 13 C NMR. Likewise, for inhibitor **BHE**, RMSD values of 2.70 for 1 H NMR, and 6.20 for 13 C NMR, and for **IHE**, RMSD values of 3.22 for 1 H NMR and 5.02 for 13 C NMR.

The low RMSD values demonstrate excellent agreement between theory and experiment, confirming the successful formation and structural integrity of the synthesized compounds.

**Table 1:** Comparison of experimental and computational NMR chemical shifts (δ, ppm) for AHE, BHE, and IHE.

Compounds	H Atoms	δ (ppm)		C Atoms	δ (ppm)	
		Experimental	Theoretical		Experimental	Theoretical
AHE	H18	7.94	8.05	C1	144.45	150.25
	H19	7.84	9.10	C2	126.18	130.87
	H20	7.84	8.54	C3	126.84	130.90
	H21	7.94	9.10	C4	129.31	132.58
	H22	7.41	9.01	C5	126.84	130.25
	H23	7.41	8.14	C6	126.18	130.99
	H24	2.27	4.25	C11	146.01	135.25
	H25	2.27	4.89	C12	13.99	17.25
	H26	2.27	5.02	C15	173.58	180.27
	H27	10.58	11.28	C16	21.37	28.42
	H28	2.26	5.14	–	–	–
	H29	2.26	4.98	–	–	–
	H30	2.26	4.87	–	–	–
	RMSD	1.30		RMSD	5.60	
BHE	H23	7.88	10.25	C1	141.65	150.54
	H24	6.60	9.47	C2	126.20	130.87
	H25	6.60	9.88	C3	127.33	130.89
	H26	7.88	8.85	C4	134.36	140.71
	H27	7.44	10.78	C5	127.33	130.25
	H28	7.44	10.98	C6	126.20	129.46
	H29	2.42	5.18	C11	144.89	150.27
	H30	2.42	6.02	C12	14.98	17.25
	H31	2.42	5.87	C15	164.75	179.24
	H32	10.89	11.98	C16	132.10	128.58
	H33	8.03	11.22	C17	127.56	132.25
	H34	7.88	8.90	C18	128.80	134.89
	H35	5.53	8.20	C19	129.32	135.25
	H36	7.88	9.41	C20	128.80	135.58
	H37	8.03	10.69	C21	127.56	130.28
	RMSD	2.72		RMSD	6.20	
IHE	H23	8.05	12.02	C1	145.18	152.27
	H24	7.90	10.87	C2	126.22	131.11
	H25	7.90	10.52	C3	127.52	132.57
	H26	8.05	12.04	C4	141.37	150.30
	H27	7.45	10.58	C5	127.52	133.02
	H28	7.45	11.77	C6	126.22	132.41
	H29	2.47	5.24	C11	150.59	152.71
	H30	2.47	5.55	C12	15.29	18.30
	H31	2.47	5.98	C15	163.31	172.25
	H32	11.12	13.02	C16	129.32	132.45
	H33	7.82	10.57	C17	122.42	125.75
	H34	8.78	12.69	C18	155.49	158.25
	H35	8.78	12.88	C20	155.49	158.58
	H36	7.82	10.53	C21	122.42	125.87
	RMSD	3.22		RMSD	5.02	

## Results and discussion

### Quantitative structure-activity relationship (QSAR)

The genetic function approximation (GFA) method is used to create a QSAR model for 10 hydrazone derivative inhibitors^16,30–34^. This model is subsequently applied to estimate the inhibition effectiveness of three inhibitors from the same family: **AHE**, **BHE**, and **IHE**, as illustrated in Scheme 1.

### Design a QSAR model using the GFA method

**Fig. 6:**
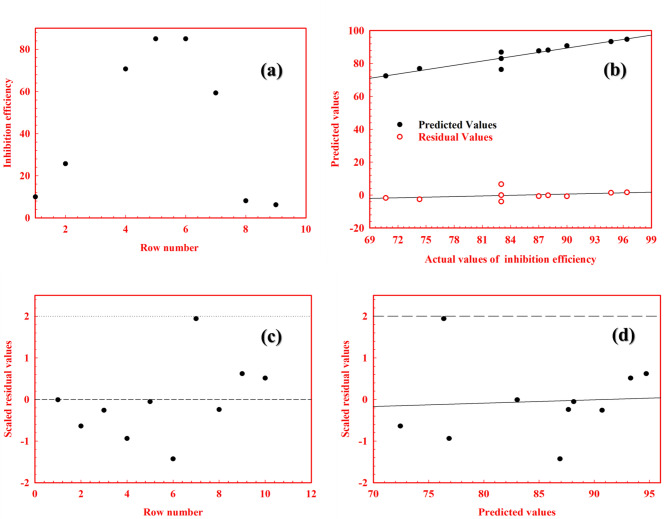
Plots of univariate analysis graph (**a**), predicted corrosion inhibition efficiency and residuals values versus measured corrosion inhibition (**b**), and Outlier analysis for inhibition efficiency (**c**, **d**) using model building GFA.

The GFA method is applied in statistical analysis to investigate and clarify the QSAR model. This process includes performing a univariate analysis of inhibition refer to (Table 2) and Fig. 6a, constructing a study table (Table 3) that compiles all relevant physicochemical parameters alongside the experimental inhibition efficiency for ten hydrazone derivative inhibitors, generating a correlation matrix, determining regression parameters (Table 4), validating the GFA model (Table 5), and deriving equations (Table 6) used to predict the inhibition efficiency for **AHE**,** BHE**, and **IHE** (Table 7)^35–37^. (Supplementary results).

To assess the fitness of a GFA model during the evolutionary process, various statistical measures can be utilized. The Friedman test for lack of fit (LOF) is often favored over the conventional least squares error metric. In Materials Studio, a revised version of Friedman’s initial equation^38^ is applied to determine the LOF, as demonstrated in Eq. (1)^39,40^

1 $$\:LOF=\:\frac{SSE}{{(1-\frac{c+dp}{M})}^{2}}$$

**Table 2:** Univariate analysis of the Inhibition data.

Statistical parameters	
Number of sample points	10
Range	25.70
Maximum	96.40
Minimum	70.70
Mean	85.01
Median	85
Variance	59.30
Standard deviation	8.1174
Mean absolute deviation	6.210
Skewness	− 0.306
Kurtosis	− 1.122

**Table 3:** Descriptors for the studied hydrazone derivatives molecules calculated using quantum chemical calculations method.

Structures	efficiency inhibition	Total energy (DMol3 Molecular)	Binding energy (DMol3 Molecular)	HOMO energy (DMol3 Molecular)	LUMO energy (DMol3 Molecular)	LUMO-HOMO energy (DMol3 Molecular)	Total dipole (DMol3 Molecular)	Dipole x (DMol3 Molecular)	Dipole y (DMol3 Molecular)	Dipole z (DMol3 Molecular)	Molecular area (vdW area) (Spatial Descriptors)	Molecular volume (vdW volume) (Spatial Descriptors)	GFA Eq. 1: prediction for B : efficiency inhibition
ABNH^16^	83.00	− 868.004	− 7.326	− 0.164	− 0.106	0.058	0.604	0.057	0.493	− 0.344	310.104	250.187	83.01
PHD-Br^30^	88.00	− 3672.157	− 9.905	− 0.178	− 0.091	0.087	2.261	− 1.468	1.648	− 0.492	418.449	347.370	88.14
PHD-H^30^	83.00	− 1102.038	− 9.980	− 0.167	− 0.079	0.088	1.645	1.204	− 1.118	− 0.071	396.064	329.470	76.37
HYD-1^31^	90.00	− 1290.438	− 10.911	− 0.162	− 0.076	0.086	1.678	0.080	1.273	− 1.090	437.082	362.965	90.71
HBNH^32^	70.70	− 809.941	− 6.191	− 0.177	− 0.101	0.076	0.716	− 0.645	0.281	0.132	260.347	212.237	72.44
MBNH^32^	74.30	− 774.151	− 6.505	− 0.179	− 0.103	0.076	0.444	− 0.436	− 0.081	0.030	272.217	221.407	76.85
PNH^34^	96.40	− 713.084	− 5.133	− 0.186	− 0.129	0.057	1.200	0.875	− 0.449	− 0.687	264.205	209.291	94.70
TNH^34^	94.70	− 1055.341	− 5.240	− 0.187	− 0.107	0.080	0.324	− 0.279	− 0.154	− 0.060	238.872	195.772	93.29
PHD-Cl^33^	83.00	− 1560.216	− 9.951	− 0.172	− 0.086	0.086	2.160	− 1.444	1.457	− 0.676	414.439	343.339	86.89
PHD-OH^33^	87.00	− 1176.773	− 10.190	− 0.164	− 0.074	0.090	1.620	1.095	− 1.106	− 0.452	406.702	336.769	87.66

**Table 4:** Correlation matrix of the studied variables.

	B : efficiency inhibition	C : Total energy (DMol3 Molecular)	D : Binding energy (DMol3 Molecular)	E : HOMO energy (DMol3 Molecular)	F : LUMO energy (DMol3 Molecular)	G : LUMO-HOMO energy (DMol3 Molecular)	H : Total dipole (DMol3 Molecular)	I : Dipole x (DMol3 Molecular)	J : Dipole y (DMol3 Molecular)	K : Dipole z (DMol3 Molecular)	L : Molecular area (vdW area) (Spatial Descriptors)	M : Molecular volume (vdW volume) (Spatial Descriptors)
B : efficiency inhibition	1.000	− 0.175	− 0.003	− 0.209	− 0.166	− 0.079	0.218	0.282	− 0.032	− 0.577	0.116	0.108
C : Total energy (DMol3 Molecular)	− 0.175	1.000	0.486	0.013	− 0.299	− 0.442	− 0.668	0.552	− 0.589	0.254	− 0.550	− 0.554
D : Binding energy (DMol3 Molecular)	− 0.003	0.486	1.000	− 0.765	− 0.908	− 0.719	− 0.816	0.022	− 0.282	0.507	− 0.986	− 0.989
E : HOMO energy (DMol3 Molecular)	− 0.209	0.013	− 0.765	1.000	0.753	0.314	0.361	0.261	0.058	− 0.347	0.698	0.693
F : LUMO energy (DMol3 Molecular)	− 0.166	− 0.299	− 0.908	0.753	1.000	0.862	0.580	0.039	0.086	− 0.224	0.835	0.850
G : LUMO− HOMO energy (DMol3 Molecular)	− 0.079	− 0.442	− 0.719	0.314	0.862	1.000	0.559	− 0.144	0.079	− 0.055	0.666	0.691
H : Total dipole (DMol3 Molecular)	0.218	− 0.668	− 0.816	0.361	0.580	0.559	1.000	− 0.163	0.378	− 0.610	0.887	0.883
I : Dipole x (DMol3 Molecular)	0.282	0.552	0.022	0.261	0.039	− 0.144	− 0.163	1.000	− 0.840	− 0.008	− 0.046	− 0.054
J : Dipole y (DMol3 Molecular)	− 0.032	− 0.589	− 0.282	0.058	0.086	0.079	0.378	− 0.840	1.000	− 0.459	0.335	0.333
K : Dipole z (DMol3 Molecular)	− 0.577	0.254	0.507	− 0.347	− 0.224	− 0.055	− 0.610	− 0.008	− 0.459	1.000	− 0.605	− 0.587
L : Molecular area (vdW area) (Spatial Descriptors)	0.116	− 0.550	− 0.986	0.698	0.835	0.666	0.887	− 0.046	0.335	− 0.605	1.000	0.999
M : Molecular volume (vdW volume) (Spatial Descriptors)	0.108	− 0.554	− 0.989	0.693	0.850	0.691	0.883	− 0.054	0.333	− 0.587	0.999	1.000

The effectiveness of the QSAR model is measured by the lack-of-fit (LOF) score, determined using Friedman’s approach, as illustrated in (Table 5). A smaller LOF score implies a reduced likelihood that the data will conform to the GFA model. The main regression analysis is assessed through the F-test, where a larger F value reflects improved model performance. Although R-squared is high, the lower cross-validated R-squared reflects the more stringent nature of cross-validation and may be influenced by the limited sample size within the model’s applicability domain.

**Table 5:** Validation table of the genetic function approximation, GFA.

	Equation 1
Friedman LOF	33.441
R-squared	0.874
Adjusted R-squared	0.774
Cross validated R-squared	0.596
Significant Regression	Yes
Significance-of-regression F-value	8.700
Critical SOR F-value (95%)	5.291
Replicate points	0.000
Computed experimental error	0.000
Lack-of-fit points	5.000
Min expt. error for non-significant LOF (95%)	2.575

Figure 6b illustrates the relationship between the experimentally measured and model-predicted corrosion inhibition efficiencies for the inhibitors under study, as derived from the model in (Table 7). It also displays the residual value distribution, highlighting a strong correlation and demonstrating the model’s robust performance.

In Fig. 6c and d, the QSAR model is assessed for possible outliers, which are identified as data points that differ from the average of the residual values by more than two standard deviations. Figure 6c displays the residual values plotted against the row number in Table 3, while Fig. 6d shows them plotted against the predicted corrosion inhibition efficiency. The dashed lines in these figures indicate the critical limit of two standard deviations, beyond which data points are deemed outliers. A review of Fig. 6c and d reveals that the QSAR model is appropriate, as no data, points exceed the dashed lines.

**Table 6:** Equation used to calculate the predicted Inhibition efficiency.

Equation	Definitions
“Y = 54.618666076 * X1 + 1126.224975122 * X3 − 21.265936346 * X4 + 1.519486196 * X5 + 143.985188778”	“X1 : D : Binding energy (DMol3 Molecular) X3 : F : LUMO energy (DMol3 Molecular) X4 : H : Total dipole (DMol3 Molecular) X5 : L : Molecular area (vdW area) (Spatial Descriptors)”

### The validation of the QSAR model

The QSAR model was utilized to assess the inhibition effectiveness of the **AHE**,** BHE**, and **IHE** inhibitors, resulting in impressive inhibition rates of 76.50%, 80.20%, and 86.99%, respectively, through the genetic function approximation technique (Table 7), presents the descriptors for the predicted **AHE**, **BHE**, and **IHE** inhibitor molecules, derived from quantum chemical calculations.

The predictive accuracy was further quantified using error-based metrics. The mean absolute error (MAE) and root mean square error (RMSE), calculated from experimental and predicted inhibition efficiencies, were 3.93 and 4.45, respectively, indicating reasonable predictive accuracy relative to the experimental data range.

Finally, No independent external validation set was used in this study. This limitation is primarily due to the small number of compounds available with complete experimental inhibition-efficiency data obtained under consistent and comparable experimental conditions. Consequently, the generalizability of the present QSAR model beyond the studied chemical space cannot be claimed and should be interpreted with caution. Model performance was therefore assessed using internal validation (cross-validation) together with complementary error-based metrics. In addition to Q² (cross-validated R² = 0.596), the predictive error was quantified using the mean absolute error (MAE) and the root mean square error (RMSE), calculated from the differences between experimental and predicted inhibition efficiencies. These error metrics provide an interpretable estimate of prediction uncertainty in the original experimental units and help contextualize the cross-validation results.

Given the limited dataset size (*n* = 10) and the absence of external validation, the QSAR model is presented as an exploratory tool intended to identify influential molecular descriptors and rationalize observed structure–activity trends, rather than as a definitive predictive model. Accordingly, conclusions drawn from the QSAR analysis are restricted to the model’s applicability domain defined by the studied compounds, and future work will focus on expanding the dataset under standardized experimental conditions to enable robust external validation.

The QSAR predictions obtained from the model were corroborated through various electrochemical assessments, including electrochemical impedance spectroscopy (EIS) and potentiodynamic polarization (PDP).

**Table 7:** Descriptors for the predicted **AHE**,** BHE**, and **IHE** molecules calculated using quantum chemical calculations and molecular dynamics simulation methods using GFA.

Structures	efficiency inhibition	Total energy (DMol3 Molecular)	Binding energy (DMol3 Molecular)	HOMO energy (DMol3 Molecular)	LUMO energy (DMol3 Molecular)	LUMO-HOMO energy (DMol3 Molecular)	Total dipole (DMol3 Molecular)	Dipole x (DMol3 Molecular)	Dipole y (DMol3 Molecular)	Dipole z (DMol3 Molecular)	Molecular area (vdW area) (Spatial Descriptors)	Molecular volume (vdW volume) (Spatial Descriptors)	GFA Eq. 1: prediction for B : efficiency inhibition
AHE	73.29	− 1169.383	− 5.786	− 0.186	− 0.104	0.082	2.404	− 2.059	− 0.966	− 0.781	273.978	214.778	76.50
BHE	86.97	− 1359.433	− 7.545	− 0.184	− 0.106	0.079	2.758	− 2.407	− 1.268	− 0.452	333.933	270.602	80.20
IHE	88.79	− 1375.408	− 7.332	− 0.190	− 0.122	0.068	1.807	0.153	− 1.801	− 0.013	324.168	264.776	86.99

### AC and DC measurements

#### AC measurements

**Fig. 7:**
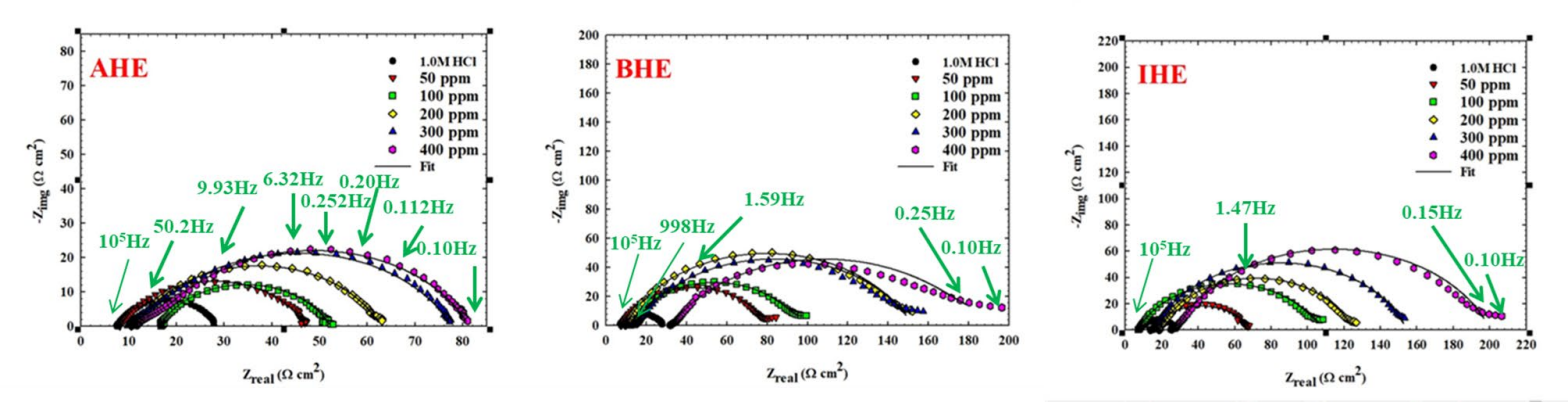
Nyquist plots of carbon steel obtained by EIS measurement in a 1.0 M HCl solution without and with different concentrations of **AHE**,** BHE**,** and IHE** inhibitors at 298 K.

**Fig. 8:**
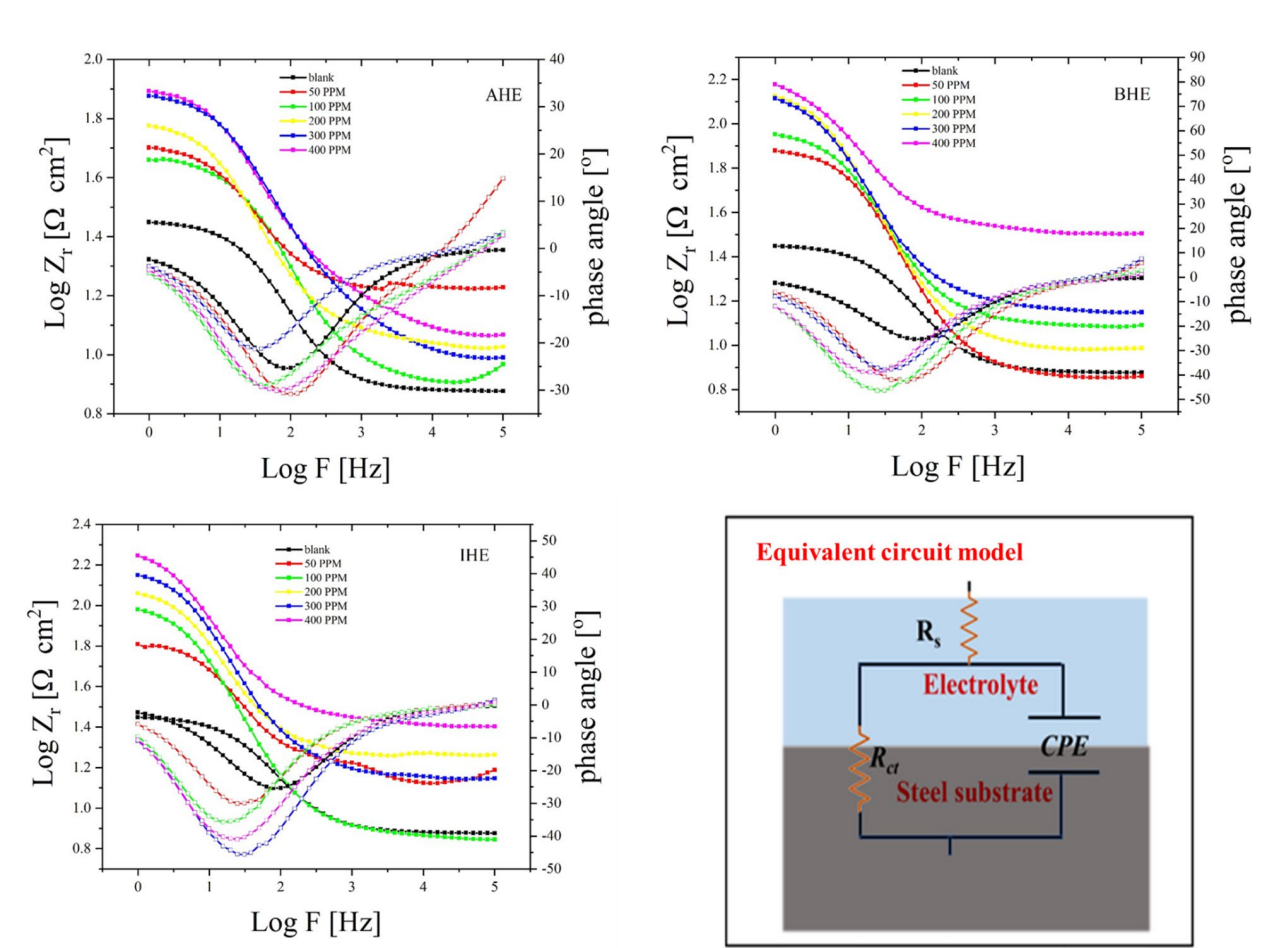
Bode plots for carbon steel corrosion in a 1.0 M HCl solution without and with different concentrations of **AHE**,** BHE**,** and IHE** inhibitors at 298 K, the equivalent circuit model used for fitting Nyquist plots.

The electrochemical kinetics, besides the capacitive performance of N80CS in 1.0 M HCl acid solutions, with and without the addition of different ppm of **AHE**, **BHE**, and **IHE** inhibitors, was examined using EIS at room temperature. Figures 7 and 8 display the plots of Nyquist and Bode for CS corrosion under acidic conditions measured by EIS, both with and without varying concentrations of **AHE**, **BHE**, and **IHE** inhibitors. The impedance bands obtained were analyzed via fitting the experimental data to the simplified electrical representation displayed in Fig. 8, with the parameters of EIS (Table 8), in detail. The Nyquist bands for the CS electrode with inhibitor concentrations are characterized by depressed single semicircles in the high-frequency range along the real axis, each with one time constant at their bases. This phenomenon can be explained by variations in surface morphology, such as roughness and other inconsistencies^41^.

The addition of **AHE**, **BHE**, and **IHE** inhibitors to the corrosive media results in a significant increase in the diameters of the semicircle. This suggests that these modifications may inhibit the N80CS corrosion submerged in a 1.0 M HCl solution, likely due to a protective barrier formation at the carbon steel interface that greatly impedes the corrosion processes^42^.

The semicircle’s larger diameter upon evaluation indicates the **AHE**, **BHE**, and **IHE** inhibitors demonstrate superior inhibitory action. Additionally, the widths of the capacitive loops increase notably with higher concentrations of the **AHE**, **BHE**, and **IHE** inhibitors, although the shape of the circles remains consistent. As the concentration of the **AHE**, **BHE**, and **IHE** inhibitors increases, the angles of phase at the frequency range middle shift to more -ve values, plus the impedance modulation at small frequency grows. This indicates a development of protective films at carbon steel/1.0 M HCl solution interfaces. To achieve a more precise and representative fit, the CPE, as shown in Fig. 5, used a constant phase element in place of the double-layer capacitance (C_dl_). Equation (2) expresses the CPE^43^.

2 $$\:{Z}_{CPE}={Y}_{0}^{-1}{\left(j\omega\:\right)}^{-n}$$

Here, j = $\:\sqrt{-1}$ refers to the imaginary unit, and *Y*_0_ denotes the CPE factor. The angular frequency is represented as in (rad s^− 1^) *ω* = 2π*f* and the *n* refers to the phase shift that is indicative of the surface inhomogeneity degree. The CPE can characterize resistance (Y_o_ = R), capacitance (Y_o_ = C), inductance (Y_o_ = L), and Warburg impedance (Y_o_ = W) for *n* = 0, 1, and 0.5, respectively. The n lower value (Table 8) following the carbon steel’s immersion in 1.0 M HCl solutions indicates that surface inhomogeneity is caused by roughening of the N80CS surface due to corrosion. The **AHE**,** BHE**,** and IHE** inhibitors adsorption has reduced surface inhomogeneity, as seen by the rise in n values when the inhibitors for **AHE**,** BHE**,** and IHE** were added.

Conversely, Fig. 7 shows that the diameter of the capacitive loop arcs for N80CS is smaller without the presence of inhibitors. Consequently, when **AHE**, **BHE**, and **IHE** inhibitors are present, the CS electrode/1.0 M HCl system exhibits increased impedance. This behavior indicates that the corrosion resistance of the metal improves due to corrosion rate reduction when **AHE**, **BHE**, and **IHE** inhibitors are used, and with greater doses of the inhibitor, this action becomes more pronounced. The following formula is used to get the double-layer capacitance$\:\left({C}_{dl}\right):$

3 $$\:{C}_{dl}={Y}_{0}{\left({\omega\:}_{m}^{n}\right)}^{n-1}$$

where the angular frequency at the imaginary part of the impedance spectrum’s highest value is represented by the symbol $\:{\omega\:}_{m}^{n}.$

Alternatively, Eq. (4) may be used to get the double-layer capacitance (C_dl_) at the frequency (fmax) where the imaginary component of the impedance spectrum is at its highest. ^44,45^

4 $$\:{C}_{dl}=\:\frac{1}{2\pi\:{f}_{max}{R}_{ct}}$$

Table 8 lists the C_dl_ values. As shown, the C_dl_ values decline with growing inhibitor concentrations, reaching a minimum at 50 ppm M.

This reduction can be ascribed to either a decline in the local dielectric constant of the film or a rise in the thickness of the protecting layer. Consequently, these values may change, and the number of active sites may decrease if some of the water molecules on the carbon steel surface are partially replaced by hydrazone derivative molecules. Additionally, the values of n (Table 8) that are close to unity when hydrazone derivative inhibitor molecules further confirm the formation of an adsorbed layer of these inhibitors on the carbon steel/solution interface.

The inhibition of corrosion efficacy value (η_EIS_%) is calculated as follows^46,47^:

5 $$\:{{\upeta\:}}_{\mathrm{E}\mathrm{I}\mathrm{S}}\mathrm{\%}=(1-\frac{{R}_{P}^{^\circ\:}}{{R}_{P}})\times\:100$$

where the polarization resistances are denoted by $\:{R}_{P}^{^\circ\:}$ and $\:{R}_{P}$, respectively, when prepared hydrazone derivative molecules are absent and present^48^.

Table 8 exhibits that the inhibition efficacy values of the **AHE**, **BHE**, and **IHE** inhibitors, respectively, are 73.29%, 86.97%, and 88.79% at 400 ppm.

**Table 8:** Electrochemical parameters derived from fitted EIS measurements for carbon steel in a 1.0 M HCl solution in the absence and presence of different concentrations of **AHE**, **BHE**, and **IHE** inhibitors at 298 K.

	Concentration (ppm)	Rs (Ru) (Ω cm^2^)	(Rp) (Ω cm^2^)	Y_o_ (µ Ω^−1^ s^n^ cm^−2^)	n	Cdl (µF cm^−2^)	Chi- square (χ^2^)×10^−6^	θ	η_EIS_ %
Blank	–	7.50	20.04	686.1	0.85	322.00	214	–	–
AHE	50	16.42	35.90	843.2	0.75	262.79	279	0.44	44.18
	100	8.06	39.36	502.8	0.80	188.59	59	0.49	49.09
	200	9.75	71.95	663.3	0.70	180.08	354	0.72	72.15
	300	10.58	52.87	801.7	0.72	194.51	772	0.62	62.10
	400	11.81	75.02	707.5	0.66	155.90	298	0.73	73.29
BHE	50	7.10	73.13	422.5	0.87	251.29	916	0.73	72.60
	100	12.20	84.59	494.5	0.78	202.00	892	0.76	76.31
	200	14.40	138.00	407.5	0.77	172.49	494	0.85	85.48
	300	9.60	141.60	478.2	0.70	150.82	654	0.86	85.85
	400	32.13	153.80	520.4	0.66	141.70	725	0.87	86.97
IHE	50	14.18	55.09	766.6	0.74	252.17	22	0.64	63.62
	100	7.25	99.76	589.9	0.75	229.45	562	0.80	79.91
	200	18.33	107.5	458.5	0.77	186.58	294	0.81	81.36
	300	14.27	143.10	434.4	0.75	172.08	615	0.86	86.00
	400	26.17	178.80	409.2	0.70	133.42	261	0.89	88.79

Furthermore, as seen in Fig. 7, the capacitive loops’ shapes are the same whether **AHE**, **BHE**, and **IHE** inhibitors are absent or present with different concentrations. The sole difference is an increase in the loops’ radius, which suggests that the corrosion process’s mechanism remains unchanged. The presence of hydrazone derivative inhibitors only reduces corrosion by increasing surface coverage and creating an adsorbed protective layer that effectively blocks charge transfer across the N80CS/HCl interface. ^49^

Conversely, the goodness of fit $\:{\chi\:}^{2}$ values were less than 0.05, signifying that the fitted equivalent circuit closely matches the actual circuit^50^. The Bode diagrams are shown in Fig. 8. The segments that are linear at intermediate frequencies in the Bode modulus diagrams of the hydrazone derivative inhibitor solutions are more pronounced than those for corrosive solutions are. The linearity rises with increasing inhibitor concentrations, reaching a maximum at 400 ppm. Additionally, the inhibitors of hydrazone derivatives’ ability to stop corrosion from (50–400) ppm can be contrasted by measuring the low-frequency impedance modulus^51^.

Figure 8 shows how increased concentrations of hydrazone derivative inhibitors result in an increase in the impedance modulus at low frequencies. This is because the carbon steel/1.0 M HCl contact is where the **AHE**, **BHE**, and **IHE** inhibitors adsorb.

Additionally, Fig. 8 demonstrates that the phase angle value in the system of inhibited carbon steel and 1.0 M HCl reaches its maximum at a concentration of 400 ppm of **AHE**, **BHE**, and **IHE** inhibitors. This suggests that the level of protection is directly related to how well the **AHE**, **BHE**, and **IHE** inhibitor molecules adhere to the N80CS surface^52^.

#### Polarization analysis

**Fig. 9:**
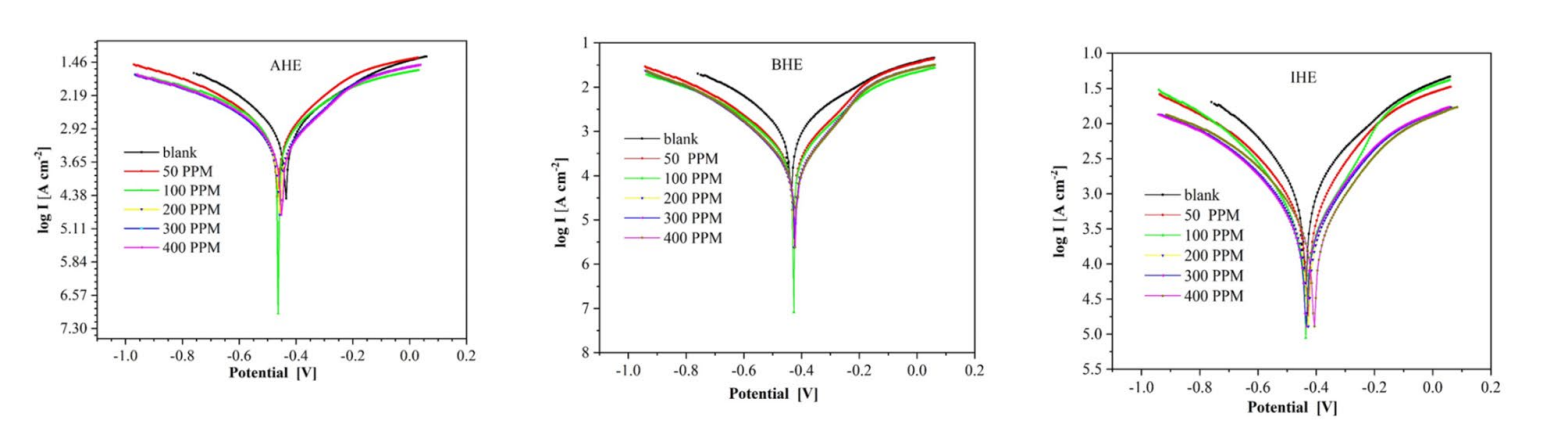
Potentiodynamic polarization curves of carbon steel into 1.0 M HCl solution in different concentrations of **AHE**,** BHE**,** and IHE** inhibitors at 298 K.

Polarization measurements were conducted to determine whether hydrazone derivatives could be categorized as anodic inhibitors, cathodic inhibitors, or mixed inhibitors and to understand how they influenced the kinetics of oxidation and reduction reactions. The effects of varying concentrations on the anodic in addition to cathodic polarization curves of synthesized **AHE**, **BHE**, and **IHE** compounds on N80CS immersed in a 1.0 M HCl solution at 298 K were investigated at a scan rate of 2.0 Mv/ s, as illustrated in Fig. 9.

The obtained electrochemical corrosion properties, such as the inhibition efficiency (η_PDP_%), corrosion current density (i_corr_), the Tafel slopes, and corrosion potential (E_corr_), are presented in Table 9. The inhibition efficacy (η_PDP_%) was computed with the following equations^53–55^.

6 $$\:{\eta\:}_{PDP}\%=\left(\frac{{i}_{corr}^{o}-{i}_{corr}}{{i}_{corr}^{o}}\right)\times\:100$$

where the variables $\:{i}_{corr}^{o}$ and $\:{i}_{corr}$ represent the density of the corrosion current (µA cm⁻²) of N80CS in a 1.0 M HCl solution in the absence and presence of synthesized **AHE**, **BHE**, and **IHE**, respectively. As given away in Fig. 9, despite the presence of **AHE**, **BHE**, and **IHE** inhibitors in different concentrations, the polarization lines in the solution of 1.0 M HCl remain nearly identical. Additionally, the current densities of both anodic and cathodic reactions on the curves of polarization have decreased, showing reduced corrosion rates. This designates that the adsorption of inhibitors molecules **AHE**, **BHE**, and **IHE** onto the sites resulted in a reduction of the N80CS electrode corrosion at the cathodic and anodic response sites. However, parallel Tafel lines observed in the curves of cathodic Tafel indicate that the hydrogen evolution reaction is activation-controlled; implying that the reduction mechanism remains unaffected by a stronger inhibitory effect and the inhibitors’ action is obstructing the metal surface. Consequently, the adsorption of hydrazone derivative molecules onto the carbon steel surface prevents the reaction via blocking the sites that are active, rather than altering the reaction process.

**Table 9:** Potentiodynamic polarization (PDP) parameters for carbon steel in a 1.0 M HCl solution in the absence and presence of different concentrations of **AHE**, **BHE**, and **IHE** inhibitors at 298 K.

	Concentration (ppm)	I_corr_ (µA/cm^2^)	E_corr_ (mV _VS_ SCE)	β_a_ (mV dec^−1^)	β_c_ (mV dec^− 1^)	K (mm/year)	$\:{\eta\:}_{PDP}\%$
Blank	–	1770	− 436	312.3	288.1	20.5669	0.00
AHE	50	1080	− 465	266.4	363.6	12.54	38.98
	100	950	− 448	314.4	383.2	11.03	46.33
	200	840	− 463	232.3	302.6	9.76	52.54
	300	710	− 455	267.4	360.7	8.25	59.89
	400	567	− 457	263.4	367.4	6.58	67.97
BHE	50	409	− 429	185.8	247.4	4.75	76.89
	100	327	− 429	189.1	219.7	3.79	81.53
	200	305	− 430	248.8	339.4	3.54	82.77
	300	278	− 428	190.4	268.1	3.23	84.29
	400	249	− 426	178.6	241.7	2.89	85.93
IHE	50	824	− 429	244.4	294.5	9.57	53.45
	100	428	− 429	242.9	276.2	4.97	75.82
	200	347	− 435	181.9	210.1	4.03	80.40
	300	317	− 432	213.6	247.6	3.68	82.09
	400	171	− 405	178.4	257.5	1.98	90.34

Additionally, Table 9 demonstrates that the values of displacement corrosion potential ($\:\varDelta\:{E}_{corr}$) for **AHE**, **BHE**, and **IHE** concentrations are intended to exploit the relationship$\:\varDelta\:{\mathrm{E}}_{\mathrm{c}\mathrm{o}\mathrm{r}\mathrm{r}}={\mathrm{E}}_{\mathrm{c}\mathrm{o}\mathrm{r}\mathrm{r}}-{\mathrm{E}}_{\mathrm{c}\mathrm{o}\mathrm{r}\mathrm{r}}^{^\circ\:}\:$, where $\:{\mathrm{E}}_{\mathrm{c}\mathrm{o}\mathrm{r}\mathrm{r}}$ and $\:{\mathrm{E}}_{\mathrm{c}\mathrm{o}\mathrm{r}\mathrm{r}}^{^\circ\:}\:$are the potential of corrosion for N80CS in the acidic medium with inhibitors and without inhibitors, respectively. For this work, the $\:\varDelta\:{\mathrm{E}}_{\mathrm{c}\mathrm{o}\mathrm{r}\mathrm{r}}$ values of the **AHE**, **BHE**, and **IHE** inhibitors indicate the mixed inhibition nature of the newly hydrazone derivatives. Consequently, **AHE**, **BHE**, and **IHE** inhibitors adsorb onto the surface of N80CS, blocking the sites of anodic and cathodic reactions, besides decreasing carbon steel dissolution in addition to hydrogen evolution by forming a barrier layer and obstructing the movement of electrons during the flow of corrosion current. Finally, it was noted that the inhibition effectiveness increased with the tendency **AHE**$\:<$**BHE**$\:<$**IHE**, where the **IHE** inhibitor shows a reduced i_corr_ in comparison to the **AHE** and **BHE** inhibitors. Thus, the **IHE** inhibitor demonstrates high adsorption onto the surface of carbon steel, achieving optimal inhibition efficiency of around 90.34% at 400 ppm. These findings corroborate the resultant impedance, displaying the identical trend: **AHE**$\:<$**BHE**$\:<$**IHE**.

Although the QSAR model captures the overall structure activity trend and provides descriptor based insight into the factors influencing inhibition efficiency, the QSAR predicted inhibition efficiencies do not always closely match the experimental values obtained from EIS and PDP measurements. This discrepancy is expected, as QSAR predictions are derived from molecular descriptors calibrated on a limited dataset, whereas electrochemical inhibition efficiencies are influenced by additional interfacial and experimental factors, such as surface coverage, adsorption strength and kinetics, protective film formation, surface heterogeneity, and methodological differences between EIS and PDP. Moreover, the relatively lower cross-validated R² (Q²) value and the absence of an independent external validation set reflect the exploratory nature of the model and limit its point-by-point predictive accuracy. Accordingly, the QSAR model should be interpreted as a supportive and mechanistic tool for rationalizing observed structure activity trends within its defined applicability domain, rather than as a definitive predictive substitute for electrochemical measurements.

### Weight loss measurements

Weight loss measurement is a method to assess corrosion resistance of N80CS immersed in a solution of hydrochloric acid with a concentration of 1.0 molar. Samples were incubated with or without **AHE**, **BHE**, and **IHE** inhibitors at various concentrations for 24 h at room temperature (298 K).

The corrosion rate (CR) is determined through Eq. (7)^56^. The protection efficiency $\:\left({{\upeta\:}}_{\mathrm{W}\mathrm{L}}\mathrm{\%}\right)$ and the associated (θ) surface coverage are calculated using Eqs. (8) and (9)^56,57^.

7 $$\:\mathrm{C}\mathrm{R}=\frac{\varDelta\:W}{At}$$

8 $$\:{{\upeta\:}}_{\mathrm{W}\mathrm{L}}\mathrm{\%}=\left(1-\frac{CR}{{CR}^{^\circ\:}}\right)\times\:100$$

9 $$\:{\uptheta\:}=\:\:\frac{{{\upeta\:}}_{\mathrm{W}\mathrm{L}}\left(\mathrm{\%}\right)}{100\:}$$

where A is the surface area of the specimen in square centimeters (cm^2^), t is the immersion time in hours, $\:{\uptheta\:}$, surface coverage of the specimen the surface, and $\:{CR}^{o}$and $\:CR$ are the corrosion rates when the studied **AHE**, **BHE**, and **IHE** inhibitors are absent and present, respectively^58^.

Table 10 shows the weight loss data for carbon steel corrosion at 298 K. It compares results without inhibitors and with various concentrations of **AHE**, **BHE**, and **IHE**.

As the concentrations of **AHE**, **BHE**, and **IHE** inhibitors increased, corrosion rates dropped. At the same time, inhibition efficiency rose, though; higher inhibitor levels enhanced the adsorption process^59^and formed a shield on the carbon steel surface. This barrier limits exposure to the harsh 1.0 M HCl solution.

### Effect of temperature

Carbon steel corrosion was studied using the weight loss method at temperatures from 298 to 358 K. Samples were placed in 1.0 M HCl for 24 h, without inhibitors and with different concentrations of **AHE**, **BHE**, and **IHE**. Table 10 presents the correlation between temperatures and the CR of N80CS without and with different concentrations (50–400 ppm) of **AHE**, **BHE**, and **IHE** inhibitors.

Evidently, as the temperature rises at a constant concentration of **AHE**, **BHE**, and **IHE**, the corrosion rate of metal also increases. Conversely, the corrosion rate consistently decreases when concentrations of **AHE**, **BHE**, and **IHE** inhibitors are added at a consistent temperature, Fig. S1. According to Table 10, when the concentration remains unchanged, increasing the temperature causes a decline in the carbon steel’s $\:{{\upeta\:}}_{\mathrm{W}\mathrm{L}}\mathrm{\%}$ in the presence of **AHE**, **BHE**, and **IHE** inhibitors.

**Table 10:** The weight loss parameters for for carbon steel in a 1.0 M HCl in the absence and presence of various concentrations of **AHE**, **BHE**, and **IHE** inhibitors at different temperatures.

Temp. K	Conc. (PPm)	AHE				BHE				IHE					
		Weight loss (mg)	CR, mg cm^− 2^ h^− 1^	θ	η_WL_(%)	Weight loss (mg)	CR, mg cm^− 2^ h^− 1^	θ	η_WL_(%)	Weight loss (mg)	CR, mg cm^− 2^ h^− 1^		θ		η_WL_(%)
298	0	400	1.19	–	–	400	1.19	–	–	400	1.19		–		–
	50	68	0.20	0.83	82.99	58	0.17	0.85	85.49	32	0.10		0.92		92.00
	100	42	0.13	0.89	89.50	53	0.16	0.87	86.74	27	0.08		0.93		93.25
	200	35	0.10	0.91	91.25	26	0.08	0.93	93.50	18	0.05		0.95		95.50
	300	27	0.08	0.93	93.25	25	0.07	0.94	93.75	12	0.04		0.97		97.00
	400	12	0.04	0.97	97.00	10	0.03	0.97	97.50	8	0.02		0.98		98.00
318	0	1475	4.39	–	–	1475	4.39	–	–	1475	4.39		–		–
	50	725	2.16	0.51	50.85	310	0.9226	0.79	78.98	220	0.65		0.85		85.09
	100	140	0.42	0.91	90.51	117	0.35	0.92	92.07	107	0.32		0.93		92.75
	200	130	0.39	0.91	91.19	94	0.28	0.94	93.63	90	0.27		0.94		93.90
	300	100	0.30	0.93	93.22	60	0.18	0.96	95.93	58	0.17		0.96		96.07
	400	78	0.23	0.95	94.71	47	0.14	0.97	96.81	37	0.11		0.97		97.49
338	0	2010	5.98	–	–	2010	5.98	–	–	2010	5.98		–		–
	50	2005	5.97	0.002	0.21	1979	5.89	0.02	1.51	693	2.06		0.66		65.51
	100	1025	3.05	0.49	48.99	820	2.44	0.59	59.19	451	1.34		0.78		77.55
	200	801	2.38	0.60	60.13	542	1.61	0.73	73.03	247	0.74		0.88		87.71
	300	495	1.47	0.75	75.36	247	0.74	0.88	87.71	195	0.58		0.90		90.30
	400	230	0.68	0.89	88.55	200	0.60	0.90	90.05	172	0.51		0.91		91.44
358	0	2200	6.55	–	–	2200	6.55	–	–	2200	6.55		–		–
	50	2192	6.52	0.004	0.40	2189	6.51	0.01	0.54	2120	6.31	0.04		3.67	
	100	2178	6.48	0.01	1.04	2168	6.45	0.01	1.49	2045	6.09	0.07		7.08	
	200	2021	6.01	0.08	8.17	2008	5.98	0.09	8.76	1637	4.87	0.26		25.62	
	300	1730	5.15	0.21	21.39	1527	4.54	0.31	30.62	745	2.22	0.66		66.15	
	400	1500	4.46	0.32	31.84	1389	4.13	0.37	36.89	621	1.85	0.72		71.78	

Thermodynamic and activation parameters were examined. They offered an understanding of the inhibition and adsorption mechanisms. The relationship between the tested temperatures and the resulting corrosion rates (CR) was evaluated using the Arrhenius equation, Eq. (10), and the transition state theory equation, Eq. (11). In these models, $\:{E}_{a}^{*}$ represents the activation energy, while ∆S* and ∆H* stand for activation entropy and enthalpy, respectively. The symbol A denotes the pre-exponential factor, and R is the universal gas constant (8.314 J/ mol K). T refers to the absolute temperature in Kelvin, while N_A_ and h represent Avogadro’s number and Planck’s constant, respectively.

10 $$\:\mathrm{log}Rate=-\frac{{E}_{a}^{*}}{2.303RT}+logA$$

11 $$\:log\frac{Rate}{T}=log\frac{R}{{N}_{A}h}+\frac{\varDelta\:{S}^{*}}{2.303R}-\frac{\varDelta\:{H}^{*}}{2.303RT}$$

Figure S2 shows Arrhenius and transition-state plots for carbon steel corrosion with and without (50–400 ppm) **AHE**, **BHE**, and **IHE**. As depicted in Figure S2, the performance of **AHE**, **BHE**, and **IHE** inhibitors aligns with the Arrhenius plots. The high regression coefficients suggest a strong fit between the experimental data and the Arrhenius equations.

The values of$\:{\:E}_{a}^{*}$, ∆H*, and ∆S* were calculated and formulated in Table 10. The activation energy (Eₐ) values calculated from the Arrhenius plots provide insight into the kinetic behavior of the corrosion process in the presence of the investigated inhibitors (**AHE**,** BHE**, and **IHE**). For all inhibitors, Eₐ increases progressively with increasing inhibitor concentration compared to the uninhibited system, indicating that the inhibitors act by increasing the energy barrier for the corrosion reaction. This behavior suggests that the adsorption of inhibitor molecules on the metal surface hinders charge transfer and slows the corrosion process.

It is apparent from Table 11 the values of apparent activation energies $\:{\:E}_{a}^{*}$for the metal deterioration are higher during contact with the **AHE**, **BHE**, and **IHE** inhibitors. The effects increased as concentrations rose, compared to none. This observation indicates the occurrence of a physicochemical process on the tested steel surface, signifying an energy barrier is created when **AHE**, **BHE**, and **IHE** inhibitors are introduced^60^. Furthermore, Table 11 shows that, in the presence of inhibitors, the positive values of $\:\varDelta\:{H}^{*}$ in our study suggest that the corrosion. It requires energy to proceed^58^. Additionally, the ∆S* values are negative, representing an association between carbon steel and the inhibitors, not dissociation. This implies that the interaction becomes more ordered. As a result, the addition of **AHE**, **BHE**, and **IHE** leads to stronger protection of the carbon steel surface. Moreover, the heat of adsorption for **AHE**, **BHE**, and **IHE** on the carbon steel surface is determined by Eq. (12) ^61^.

12 $$\:\mathrm{log}\left(\frac{\theta\:}{1-\theta\:}\right)=logA+logC-\left(\frac{{Q}_{ads}}{2.303RT}\right)\:\:\:$$

Figure S3 and Table 11 represented the calculated Q_ads_ values.

Values of $\:{Q}_{ads}$ are consistently -Ve across wholly concentrations. Additionally, as temperature increases, the inhibition efficiencies decrease, suggesting the presence of physical forces between **AHE**, **BHE**, and **IHE** molecules and the carbon steel surface^62^.

**Table 11:** Thermodynamic parameters of activation for carbon steel dissolution in a 1.0 M HCl solution without and with various concentrations of **AHE**, **BHE**, and **IHE** inhibitors.

Inhibitor name	Inhhibitor conc, ppm	linear regression equations	*R* ^2^	$\:{\:E}_{a}^{*}$ (kj mol^− 1^)	linear regression equations	*R* ^2^	∆H* (kj mol^− 1^)	∆S* (j mol^−1^k^− 1^)	linear regression equations	*R* ^2^	Q_ads_ (kj mol^− 1^)
AHE	0	y = 4.5231–1.2881x	0.8287	24.66	y = 1.5758 -1.1468 x	0.7905	21.92	-133.41			
	50	y = 8.6060–2.7091x	0.8723	51.87	y = 5.6540 -2.5665 x	0.8588	49.06	-71.18	y = -20.7774 + 6.4266 x	0.8408	-0.12
	100	y = 9.8350 − 3.2098 x	0.9783	61.46	y = 6.8868 -3.0683 x	0.9762	58.65	-52.37	y = -5.7180 + 2.0194 x	0.7517	-0.04
	200	y = 9.8706 -3.2419 x	0.9897	62.07	y = 6.9258 -3.1015x	0.9887	59.28	-51.78	y = -4.7512 + 1.7438 x	0.7958	-0.03
	300	y = 9.7876–3.2559 x	0.9957	62.34	y = 6.8387 -3.1142 x	0.9954	59.53	-53.10	y = -3.3688 + 1.3610 x	0.7958	-0.03
	400	y = 10.623–3.5985 x	0.9871	68.90	y = 7.6758–3.4573 x	0.9863	66.09	-40.33	y = -2.4291 + 1.1693 x	0.9195	-0.02
BHE	50	y = 9.3547–2.9899 x	0.9340	57.25	y = 6.4057–2.8481 x	0.9272	54.44	-59.71	y = -17.6975 + 5.5851x	0.8628	-0.11
	100	y = 9.2266–3.0173 x	0.9665	57.77	y = 6.2783 -2.8759 x	0.9634	54.97	-61.66	y = -14.8200 + 4.8424 x	0.7069	-0.09
	200	y = 10.2960–3.4168 x	0.9926	65.42	y = 7.3511 -3.2765 x	0.9921	62.63	-45.29	y = -10.8730 + 3.6917 x	0.7741	-0.07
	300	y = 9.3106–3.1501 x	0.9575	60.32	y = 6.3676 -3.0103 x	0.9541	57.54	-60.29	y = -7.3099 + 2.6387 x	0.6759	-0.05
	400	y = 10.9714–3.7442 x	0.9872	71.69	y = 8.0261 -3.6037 x	0.9864	68.88	-34.99	y = -8.2530 + 2.9996 x	0.7844	-0.06
IHE	50	y = 9.7662 − 3.1958 x	0.9915	61.19	y = 6.8195 -3.0548 x	0.9905	58.39	-53.40	y = -12.2363 + 4.0536 x	0.8157	-0.08
	100	y = 10.0270–3.3290 x	0.9943	63.74	y = 7.0800 -3.1879 x	0.9940	60.94	-49.42	y = -11.1363 + 3.7720 x	0.7433	-0.07
	200	y = 9.9506 -3.3538 x	0.9806	64.22	y = 7.0020 -3.2122 x	0.9793	61.40	-50.61	y = -8.0180 + 2.8535 x	0.7441	-0.05
	300	y = 9.1243–3.1502 x	0.9986	60.32	y = 6.1751–3.0085 x	0.9985	57.51	-63.23	y = -5.5294 + 2.1423 x	0.8906	-0.04
	400	y = 9.7005 -3.1502 x	0.9993	64.71	y = 6.7527–3.2382 x	0.9993	61.90	-54.42	y = -5.8565 + 2.2882 x	0.9296	-0.04

### Adsorption isotherm

**Fig. 10:**
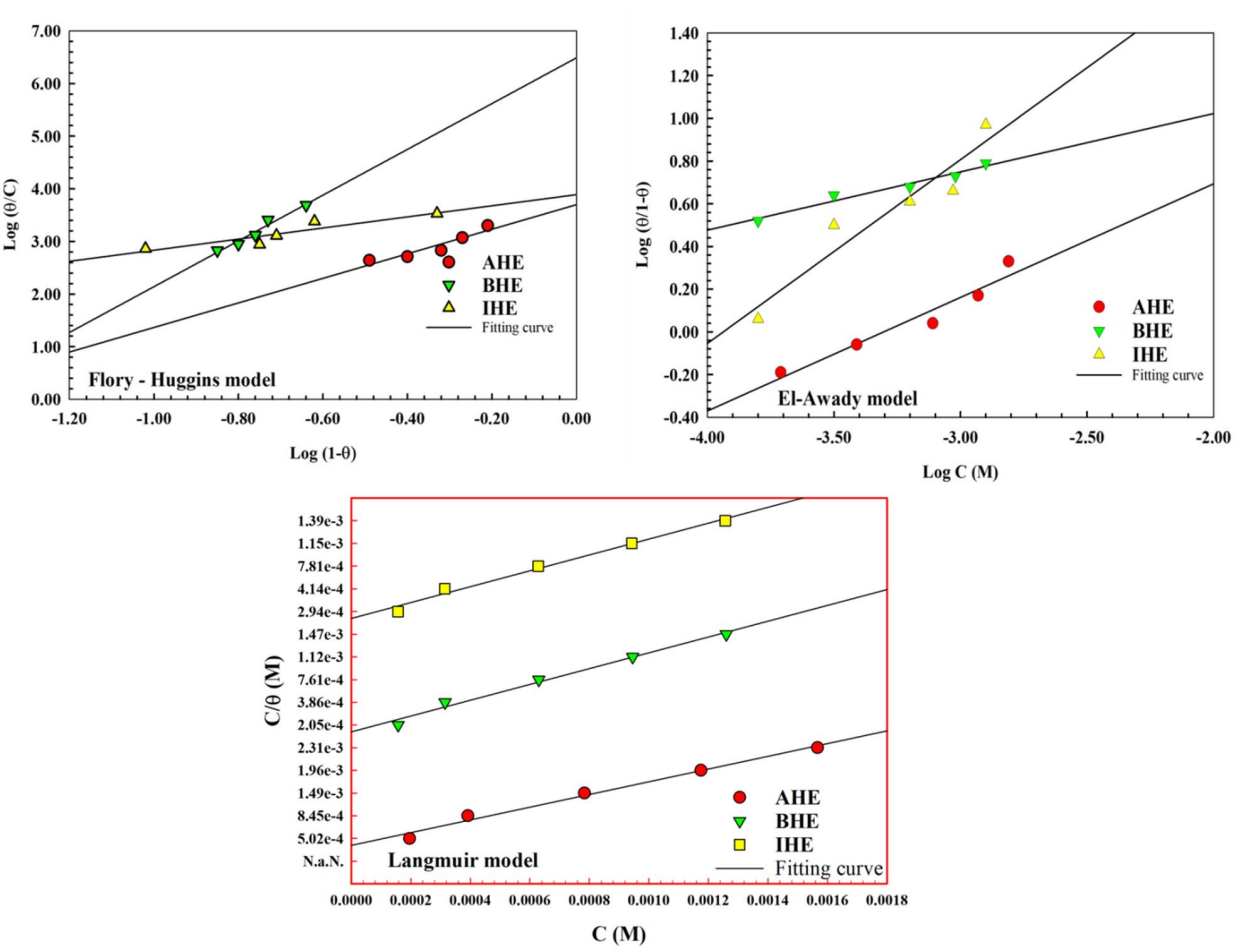
Flory-Huggins, El-Awady and Langmuir adsorption isotherm models for carbon steel in 1.0 M HCl solutions in the absence and presence of different concentrations of **AHE**,** BHE**,** and IHE** inhibitors by PDP study at 298 K.

An adsorption isotherm explains how hydrazone derivative inhibitor molecules interact with the surface of N80CS they protect. It offers information on the nature as well as the strength of the adsorption processes, which directly affects how well it resists corrosion. To explore the mechanism of hydrazone compounds adsorption on N80CS surfaces in 1.0 M HCl solutions, adsorption isotherms for corrosion inhibitors, including Langmuir, Kinetic, and Flory-Huggins, are stated at this work as shown in Fig. 10 and Eqs. (13–15)^50,63^.

13 $$\:\mathrm{L}\mathrm{a}\mathrm{n}\mathrm{g}\mathrm{m}\mathrm{u}\mathrm{i}\mathrm{r}\:\mathrm{i}\mathrm{s}\mathrm{o}\mathrm{t}\mathrm{h}\mathrm{e}\mathrm{r}\mathrm{m}\:\:\mathrm{C}/{\uptheta\:}=1/{\mathrm{K}}_{\mathrm{a}\mathrm{d}\mathrm{s}}+\mathrm{C}\:\:\:$$

14 $$\:\mathrm{E}\mathrm{l}-\mathrm{A}\mathrm{w}\mathrm{a}\mathrm{d}\mathrm{y}\:\mathrm{i}\mathrm{s}\mathrm{o}\mathrm{t}\mathrm{h}\mathrm{e}\mathrm{r}\mathrm{m}\:\:\:\mathrm{l}\mathrm{o}\mathrm{g}[{\uptheta\:}/(1-{\uptheta\:})]=\mathrm{l}\mathrm{o}\mathrm{g}{\mathrm{K}}^{{\prime\:}}+\:\mathrm{Y}\mathrm{L}\mathrm{o}\mathrm{g}\mathrm{C}$$

15 $$\:\mathrm{F}\mathrm{l}\mathrm{o}\mathrm{r}\mathrm{y}-\mathrm{H}\mathrm{u}\mathrm{g}\mathrm{g}\mathrm{i}\mathrm{n}\mathrm{s}\:\mathrm{i}\mathrm{s}\mathrm{o}\mathrm{t}\mathrm{h}\mathrm{e}\mathrm{r}\mathrm{m}\:\mathrm{l}\mathrm{o}\mathrm{g}[{\uptheta\:}/\mathrm{C}]=\mathrm{l}\mathrm{o}\mathrm{g}{\mathrm{K}}_{\mathrm{a}\mathrm{d}\mathrm{s}}+\mathrm{x}\mathrm{l}\mathrm{o}\mathrm{g}\left(1-{\uptheta\:}\right)$$

The values of surface coverage (θ) acquired from EIS are shown in Table 8 for various concentrations of inhibitors. When evaluating each model for each inhibitor, the fitting correlation coefficients for the Langmuir model are near to 1.0. This indicates that the **AHE**, **BHE**, and **IHE** inhibitors adsorption on CS corresponds well with the Langmuir adsorption isotherm^64^. The values of equilibrium constant $\:{K}_{ads}$(M^− 1^) can be determined by taking the reciprocal of the intercept. Furthermore, these equilibrium constant values are used to estimate the free energy of adsorption, $\:{{\Delta\:}G}_{ads}^{o}$ using Eq. (16).

16 $$\:{\:\:\:\:\:\:\:\:\:\:\:\:\:\:\:\:\:\:\:\:\:\:\:\:\:\:{\Delta\:}\mathrm{G}}_{\mathrm{a}\mathrm{d}\mathrm{s}}^{\mathrm{o}}=-\mathrm{R}\mathrm{T}\mathrm{l}\mathrm{n}\left(55.5{\mathrm{K}}_{\mathrm{a}\mathrm{d}\mathrm{s}}\right)$$

The equilibrium constant values $\:\:{K}_{ads}$(M^− 1^), and the Gibbs free energy of adsorption, $\:{\varDelta\:G}_{ads}^{o}$(kJ mol^− 1^) are presented in Table 12. The adsorption behavior of the investigated inhibitors (**AHE**, **BHE**, and **IHE**) was analyzed using the corresponding adsorption isotherms, as evidenced by the high linear correlation coefficients (R² ≈ 0.98–0.99), indicating good conformity of the experimental data with the applied adsorption model. The obtained adsorption equilibrium constants $\:{K}_{ads}$(M^− 1^), increase in the order **AHE** < **BHE** < **IHE**, reflecting progressively stronger adsorption of inhibitor molecules onto the metal surface. Table 12 shows that the standard free energy values of adsorption are all-negative, confirming the spontaneous nature of the adsorption process. The magnitude of $\:{\varDelta\:G}_{ads}^{o}$ ranges from − 30.05 to − 36.00 kJ·mol⁻¹, suggesting that adsorption occurs through a mixed mechanism involving both physical and chemical interactions. In particular, the more negative $\:{\varDelta\:G}_{ads}^{o}$ value observed for **IHE** is consistent with its higher $\:{K}_{ads}$ value, indicating a stronger and more stable adsorption at the CS/solution interface.

**Table 12:** Langmuir adsorption isotherm parameters for the **AHE**,** BHE**,** and IHE** inhibitors onto carbon steel in a 1.0 M HCl solution at 298 K.

Inhibitor name	linear regression equations	*R* ^2^	K_ads_ (M^− 1^)	$\:{\varDelta\:\mathrm{G}}_{\mathrm{a}\mathrm{d}\mathrm{s}}^{\mathrm{o}}$ (kj mol^− 1^)
AHE	y = 0.0003 + 1.3298x	0.98	3333	− 30.05
BHE	y = 0.0001 + 1.0374x	0.99	10,000	− 32.78
IHE	y = 2.7201E^− 05^ + 1.1504x	0.99	36,763	− 36.00

### Analysis investigations

#### AFM analysis

**Fig. 11:**
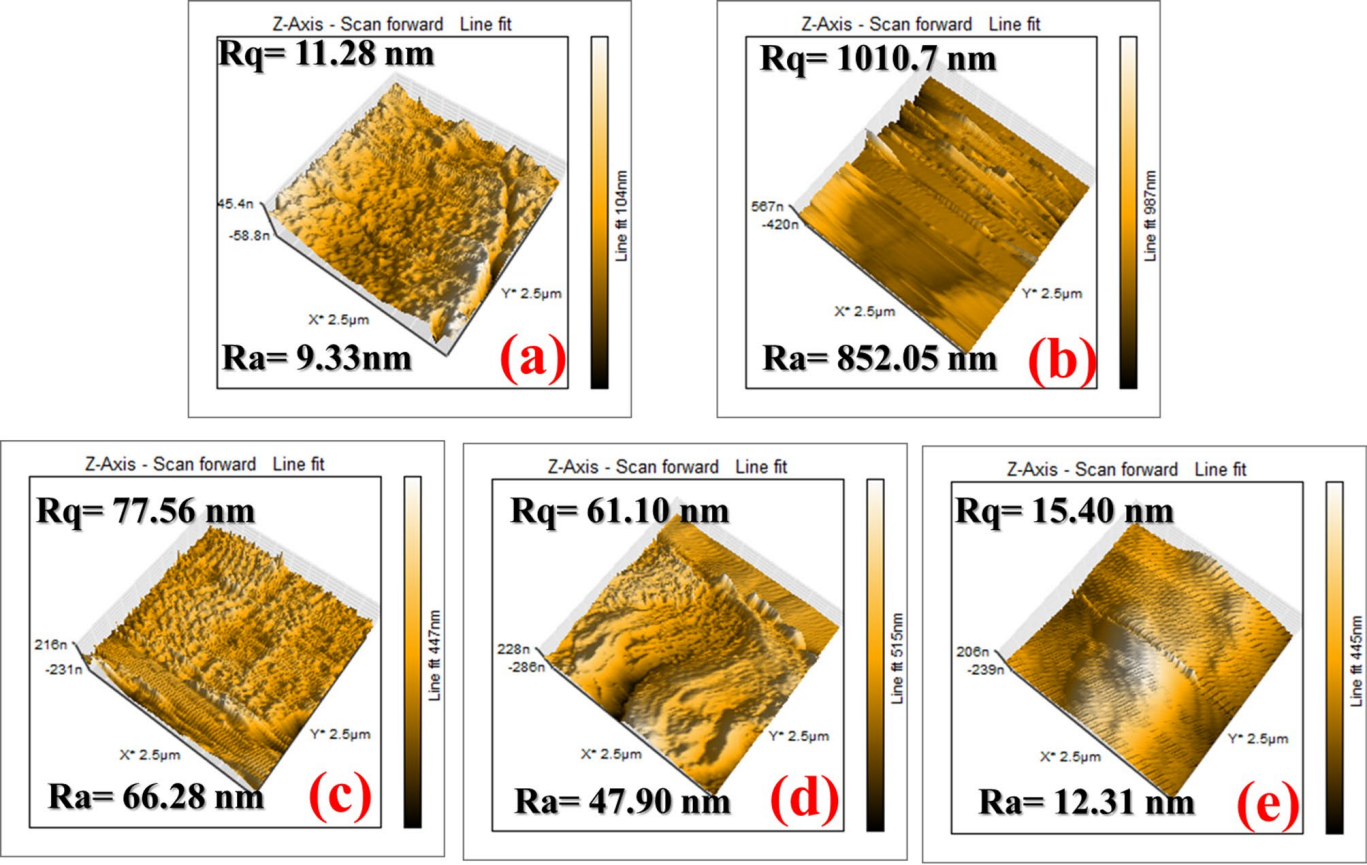
3D AFM images of (**a**) polished CS sample (**b**) after 24 h of immersion in 1 M HCl electrolyte, (**c**–**e**) after 24 h of immersion in 1.0 M HCl containing 400 ppm **AHE**,** BHE**,** and IHE** inhibitors respectively.

Atomic force microscopy (AFM) analysis can precisely evaluate the roughness of metal surfaces^65^. To determine how well the compounds under investigation inhibited corrosion, CS substrates were immersed in 1.0 M HCl solutions for a whole day both before and after 400 ppm inhibitors of AHE, BHE, and IHE were added. Figure 11 displays 3D images from the surface morphology AFM investigation. The noticeable corrosion pitting of CS is likely due to the degradation of the CS metal. However, the addition of 400 ppm **AHE**,** BHE**,** and IHE** inhibitors resulted in a uniform, more smoother, and more adhesive surface^66^. The (Ra) average roughness of the polished CS is 9.33 nm (Fig. 11a), and the CS sample in the acid solution was 852.05 nm (Fig. 11b). However, after the addition of 400 ppm **AHE**, **BHE**, and **IHE** inhibitors, the average roughness decreased to 66.28, 47.90, and 12.31 nm (Fig. 11c-e), respectively. This reduction is likely the result of the inhibitor molecules creating a barrier. Additionally, the CS surface is more effectively protected by **IHE** compared to **AHE** and **BHE** as indicated by the lower average roughness value.

### Water contact angle (WCA) measurement analysis

**Fig. 12:**
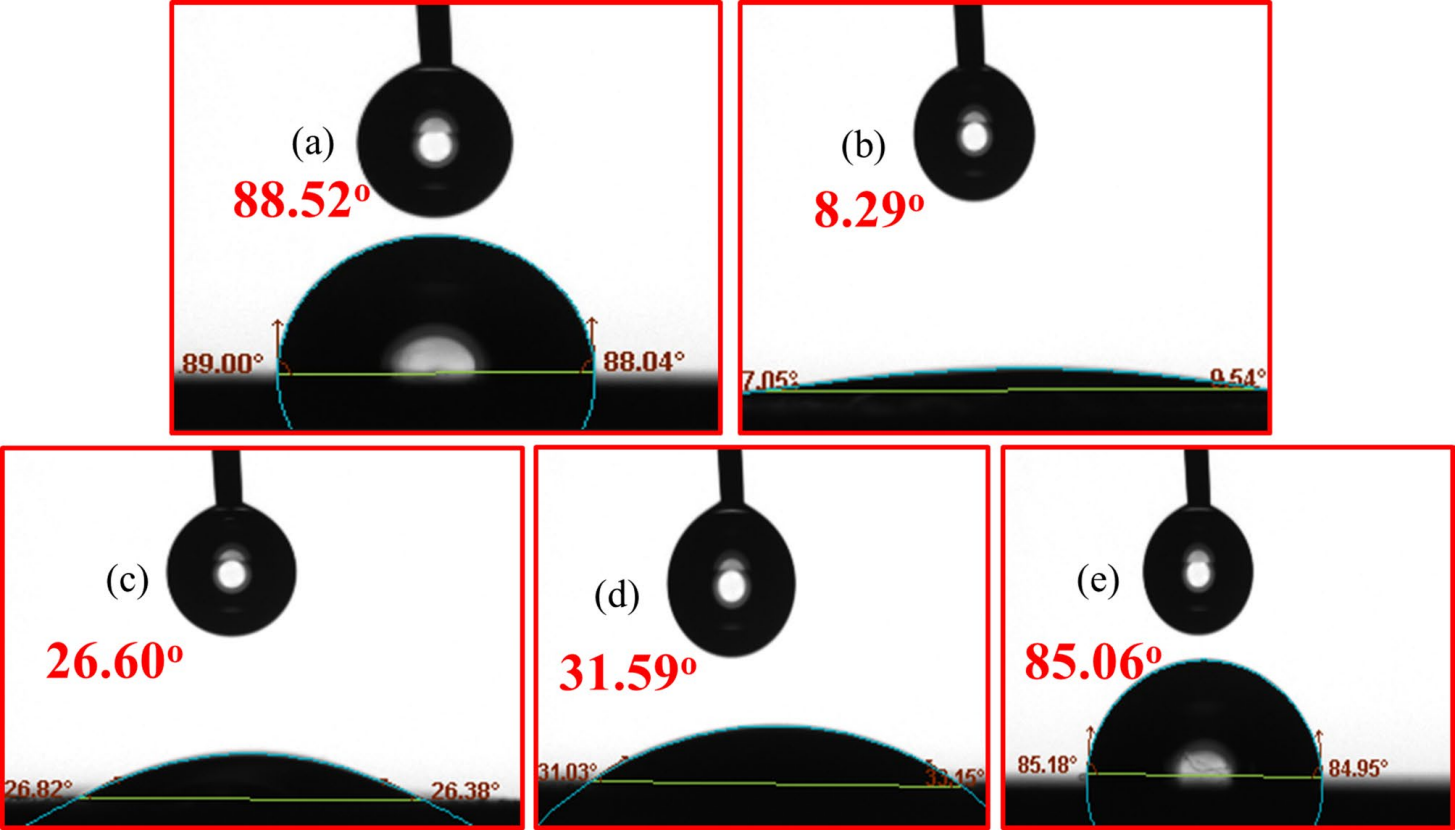
Water contact angle evaluation of (**a**) polished CS sample (**b**) after 24 h of immersion in 1 M HCl electrolyte, (**c**–**e**) after 24 h of immersion in 1.0 M HCl containing 400 ppm **AHE**,** BHE**,** and IHE** inhibitors respectively.

Surfaces are classified as hydrophobic if their contact angle (h) exceeds 90° and hydrophilic if it is below 90°^67^. Contact angle measurements were taken for CS samples immersed in 1.0 M HCl solutions for 24 h, both after and before the addition of 400 ppm of the tested corrosion inhibitors at room temperature. As shown in Fig. 12a, the polished carbon steel surface has a contact angle of 88.52°. Figure 12b illustrates the water contact angle on the N80CS surface in a 1.0 M HCl solution without inhibitors, which is 8.29°, demonstrating high hydrophilicity and promoting metal dissolution. Figure 12c-e demonstrates the water contact angles on the CS surface in 1.0 M HCl solutions with the presence of 400 ppm of the **AHE**,** BHE**, and **IHE** inhibitors. The presence of **AHE**,** BHE**, and **IHE** inhibitors results in the development of a thin film upon the corroded surface, increasing the contact angles to 26.6°, 31.59°, and 85.06° for **AHE**,** BHE**, and **IHE** inhibitors, respectively. This suggests that the surface becomes less hydrophilic and more hydrophobic, thereby reducing metal dissolution^68^.

### EDX SEM analysis

**Fig. 13:**
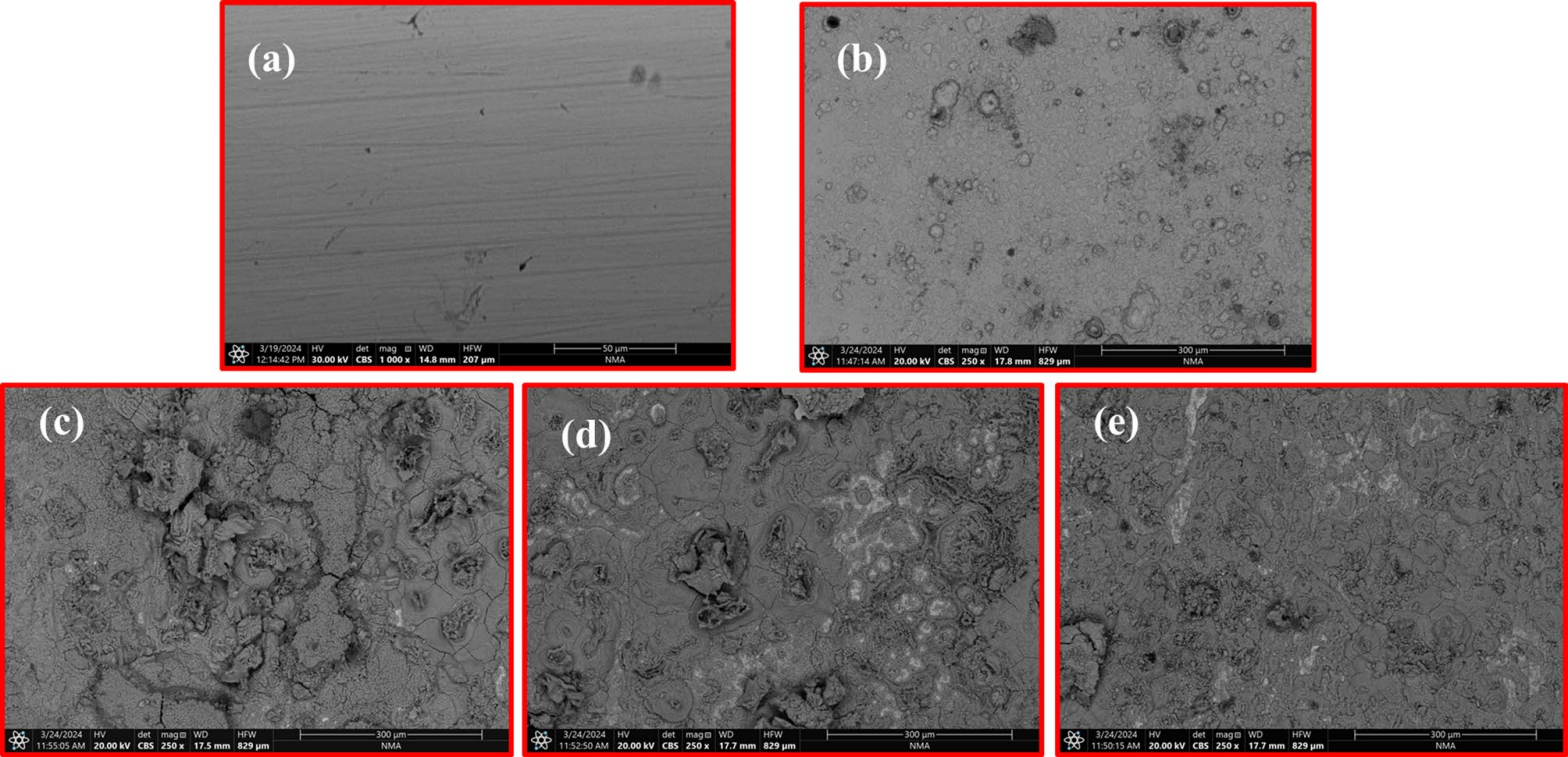
SEM images of the (**a**) polished CS sample (**b**) after 24 h of immersion in 1 M HCl electrolyte, (**c**–**e**) after 24 h of immersion in 1.0 M HCl containing 400 ppm **AHE**,** BHE**,** and IHE** inhibitors respectively.

Scanning electron micrographs (SEM) are employed for studying the CS surface morphological changes on both corroded and protected forms. Figure 13a displays an SEM image of a polished, cleaned CS surface, showing no cracks, but it is not uniform and has some scratches. Figure 13b shows an SEM image of the CS surface after dipping in a 1.0 M HCl solution for one day. The CS surface exhibits small cavities, scattered pits, significant roughness, and deposited corrosion products, indicating severe acid-induced damage and a high corrosion rate.

**Fig. 14:**
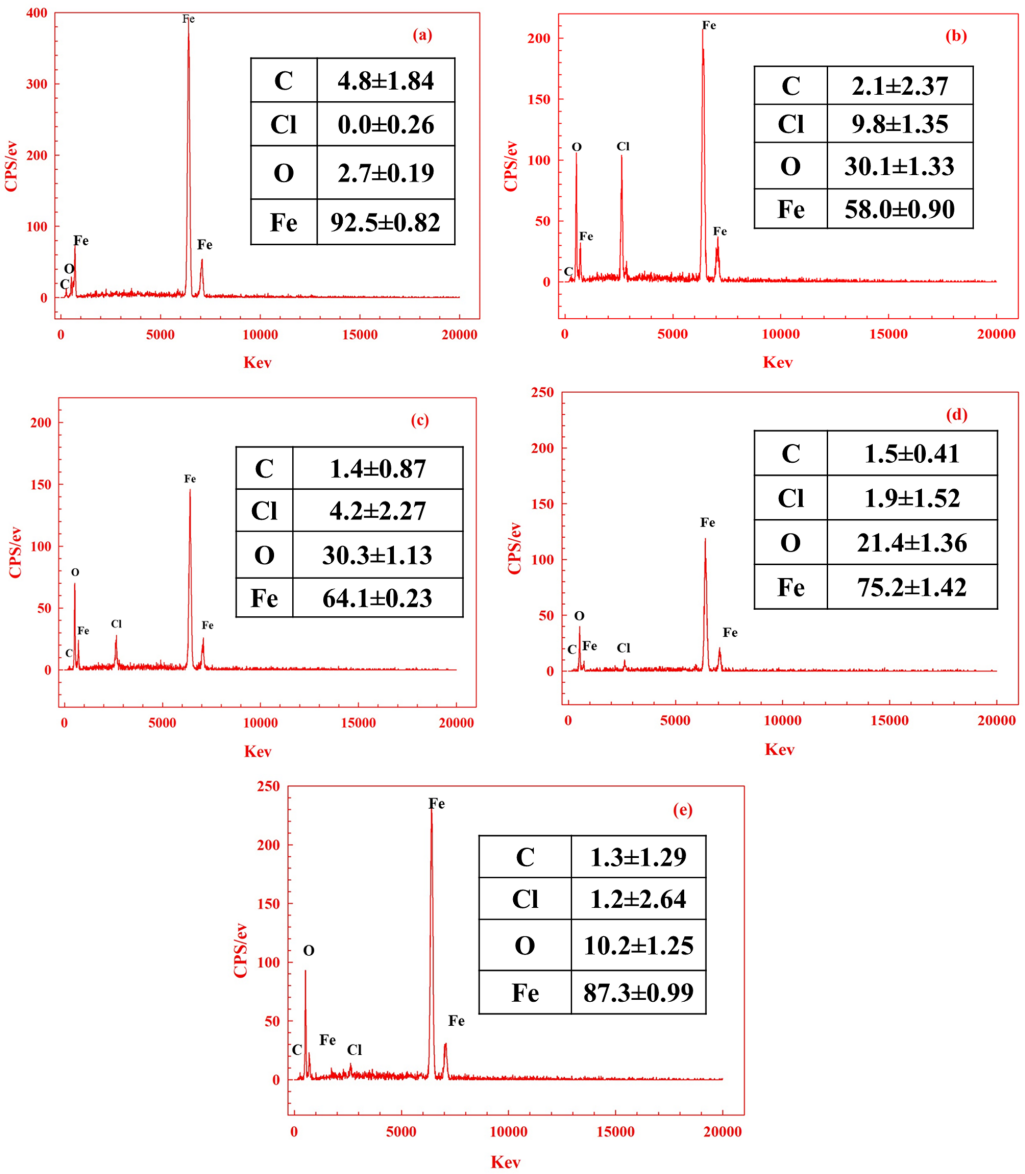
EDX analysis and atomic contents percentage of elements (mass %) obtained by EDX analysis of the (**a**) polished CS sample (**b**) after 24 h of immersion in 1 M HCl electrolyte, (**c**–**e**) after 24 h of immersion in 1.0 M HCl containing 400 ppm **AHE**,** BHE**,** and IHE** inhibitors respectively. at 298 K.

Figure 13c-e presents SEM images of CS surfaces after being immersed for 24 h in an acidic solution containing 400 ppm concentrations of **AHE**,** BHE**, and **IHE** inhibitors. These images reveal that the CS surfaces were mainly coated with **AHE**,** BHE**, and **IHE** molecules, resulting in smoother surfaces compared to Fig. 13b. Notably, the CS surface with the **IHE** inhibitor appears the smoothest, suggesting effective protection^69^. The EDX examination of the iron sample composition percentages is presented in Fig. 14, supporting the conclusion that **IHE** effectively prevents CS corrosion.

### Computational measurements

#### DFT calculations

**Fig. 15:**
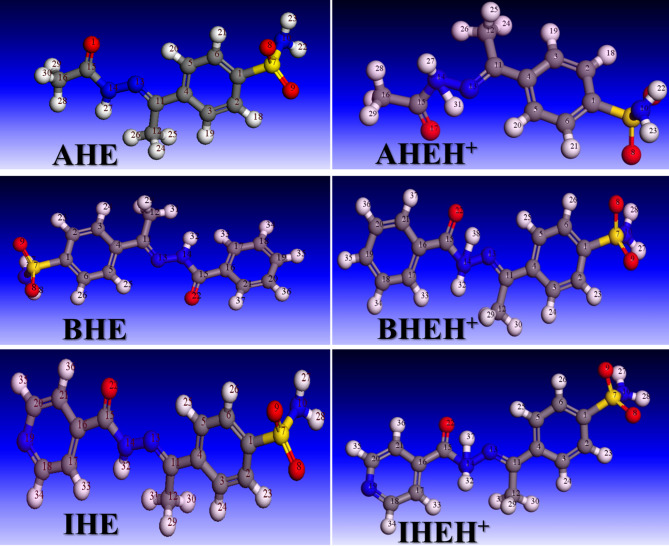
The optimized molecular structure of neutral and protonated forms of the **AHE**,** BHE**,** and IHE** inhibitors.

**Fig. 16:**
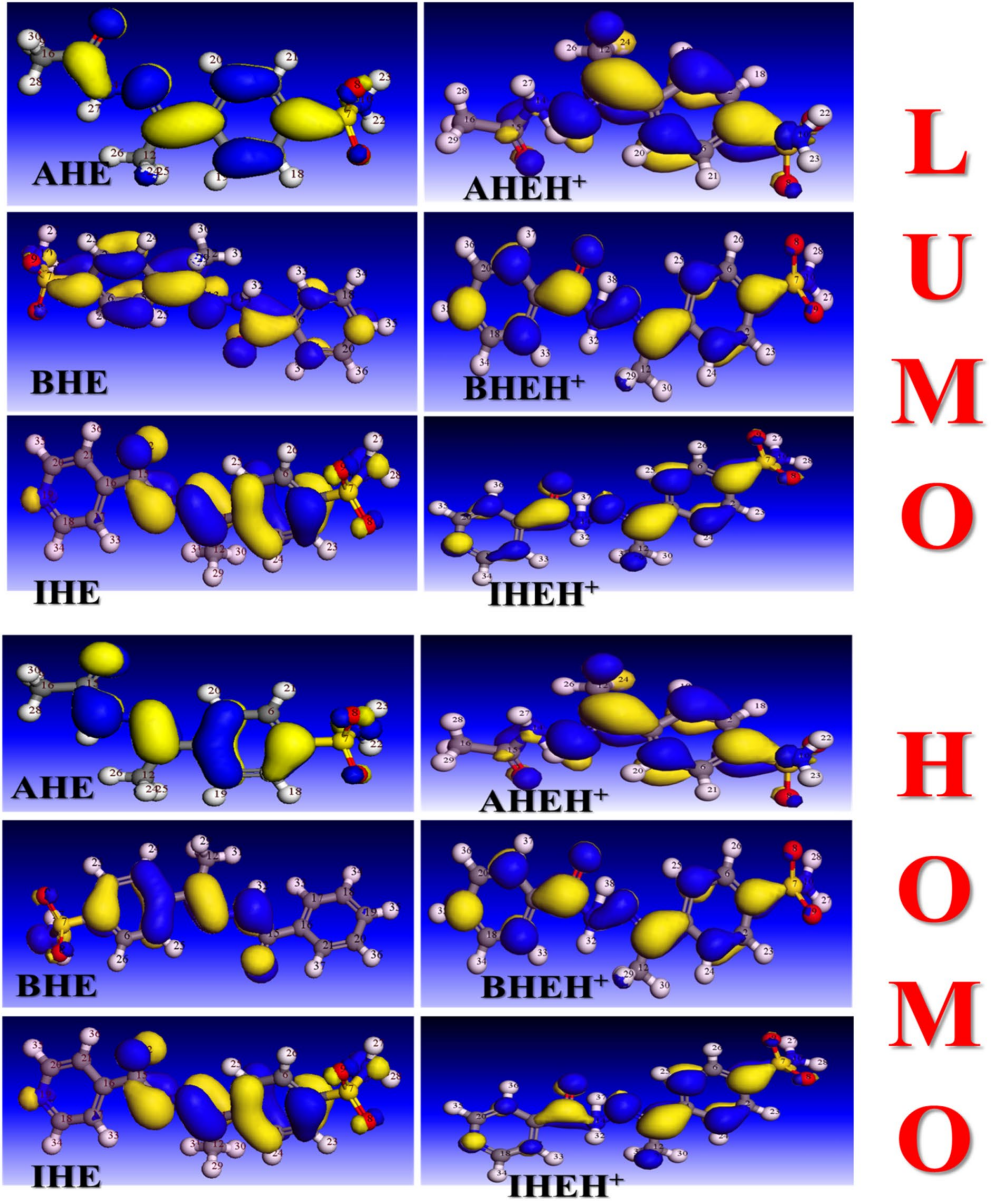
The frontier molecular orbitals of neutral and protonated forms of the **AHE**,** BHE**,** and IHE** inhibitors.

Optimised molecular structures, along with the HOMOs and LUMOs for the neutral, **AHE**,** BHE**, and **IHE** and protonated forms **AHEH**^**+**^, **BHEH**^**+**^, and **IHEH**^**+**^_,_ are shown in Figs. 15 and 16, and the calculated DFT index is provided in Table 13. HOMO characterizes the tendency of the molecules to donate electrons, while LUMO reflects the molecule’s ability to accept electrons or act as an electron acceptor in chemical interactions^70^. The gap of energy (ΔE) is determined by the energy gap between the molecules’s most stable occupied electron level and its least stable unoccupied level^71,72^.

A smaller gap of energy (ΔE) corresponds to increased molecular reactivity^73^. **IHE** exhibits a smaller ΔE compared to **AHE** and **BHE**, indicating better inhibition efficiency, consistent with experimental findings. Additional quantum chemical parameters, including electron affinity (A) and ionization energy (I), were calculated for understanding the interactions between carbon steel and the **AHE**, **BHE**, and **IHE** inhibitors^74,75^.

Electronegativity (χ)^76^ is as well examined to assess the inhibition effectiveness of the studied inhibitors for CS corrosion. The calculated χ values for **AHE**, **BHE**, and **IHE** inhibitors, shown in Table 13, suggest that the interaction between Fe and these inhibitors involves a coordinated covalent bond^77^. Softness (S), an indicator of molecular stability, is inversely related to hardness (η)^70^. This means that softer molecules are extra polarisable, have greater chemical activity, smaller kinetic stability, and offer better protection compared to harder molecules^70,78,79^. The **IHE** inhibitor demonstrates greater softness (S = 0.73) than **AHE** (S = 0.66) and **BHE** (S = 0.69). The higher corrosion inhibition efficiency of AHE, BHE, and IHE inhibitors is linked to their elevated E_HOMO_ values and reduced ΔE. The global electrophilicity index (ω) and the global nucleophilicity index (ε) are crucial factors in assessing the effectiveness of these inhibitors in preventing corrosion^77^. Consistent with Table 13, ω and ε values prove that the **IHE** inhibitor has a higher effective inhibitory activity than **AHE** and **BHE.**

Furthermore, the (ω+), electron-accepting power, and (ω−), electron-donating power (ω−), are calculated and presented in Table 13.

The results show that the **IHE** inhibitor is the most effective, as it has the maximum global electrophilicity index (ω = 7.817 eV) and electron-accepting power (ω+ = 5.676 eV) compared to **AHE** and **BHE**. Additionally, Table 13 reveals that the values of (ΔN), the fraction of transferred electrons for **AHE**, **BHE**, and **IHE** compounds, are smaller than 3.6^80,81^, indicating electron transfer between the inhibitors and the CS surface atoms. The metal/inhibitor interaction energy ($\:{{\Delta\:}{{\rm\:E}}}_{metal/inhibitor}$) values confirm the **AHE**, **BHE**, and **IHE** inhibitors are effective inhibitors for CS corrosion.

Lastly, the calculated ΔE _back−donation_ (eV) back-donation energy is less than 0 for the **AHE**, **BHE**, and **IHE** inhibitors. This specifies that energy back and donation from studied **AHE**, **BHE**, and **IHE** inhibitors molecules to the carbon steel surface are favorable.

Table 13 shows that the energy gap (ΔE) values are 0.467, 0.333, and 0.299 eV for **AHEH**^**+**^, **BHEH**^**+**^, and **IHEH**^**+**^, respectively. Consequently, the **IHEH**^**+**^ inhibitor, with the lowest ΔE value in the gas phase, is more easily adsorbed on the N80CS surface, enhancing its corrosion inhibition effectiveness, which aligns with previous experimental results. Greater adsorption of **AHEH**^**+**^, **BHEH**^**+**^, and **IHEH**^**+**^ on the CS surface is correlated with lower electronegativity, measured at 3.425, 3.731, and 3.391 eV for **AHEH**^**+**^, **BHEH**^**+**^, and **IHEH**^**+**^, respectively^82^.

Furthermore, Table 13 indicates that soft protonated molecules adhere more successfully to the CS electrode surface than hard non-protonated molecules, provided that better protection. The hardness (η) values are 0.233, 0.166, and 0.150 eV for **AHEH**^**+**^, **BHEH**^**+**^, and **IHEH**^**+**^, respectively. The electrophilicity index (ω) values are 25.11, 41.85, and 38.42 eV for **AHEH**^**+**^, **BHEH**^**+**^, and **IHEH**^**+**^, respectively, while the nucleophilicity index (ε) values are 0.040, 0.024, and 0.026 eV for **AHEH**^**+**^, **BHEH**^**+**^, and **IHEH**^**+**^, respectively. These results suggest that protonated inhibitors, with their low ω values, are effective nucleophilic inhibitors.

The (ΔE _back−donation_) is -0.058, -0.042, and − 0.037 eV for **AHEH**^**+**^, **BHEH**^**+**^, and **IHEH**^**+**^, respectively. Additionally, the values of ΔN for the **AHEH**^**+**^, **BHEH**^**+**^, and **IHEH**^**+**^ molecules are 2.667, 2.823, and 4.276 eV, respectively. Table 13 displays the interaction energies between the protonated inhibitor molecules and the CS electrode, which are 1.661, 1.325, and 2.735 eV for **AHEH**^**+**^, **BHEH**^**+**^, and **IHEH**^**+**^, respectively. This indicates that **AHE**, **BHE**, and **IHE** are adsorbed in their protonated forms.

Finally, The DFT calculations support a mixed adsorption mechanism for the studied inhibitors. The optimized molecular structures show multiple adsorption-active centers (heteroatoms and π-electron regions) capable of interacting with the metal surface. From an electronic perspective, the frontier molecular orbitals indicate that the inhibitors can donate electron density from the HOMO to vacant metal orbitals and, simultaneously, accept electron density into the LUMO through back-donation, which is consistent with a chemisorption contribution via coordination/charge-transfer interactions. In parallel, the physisorption contribution is attributed to electrostatic attraction of protonated inhibitor species to the charged metal surface (via ion-pair interactions with adsorbed anions). Therefore, the DFT-derived electronic features are fully consistent with the experimental adsorption thermodynamics, confirming that adsorption involves both physical attraction and chemical bonding.

**Table 13:** Calculated quantum chemical parameters for the neutral and protonated forms of the **AHE**,** BHE**,** and IHE** inhibitors obtained with the DFT using DMol^3^module.

Property	AHE	BHE	IHE	AHEH^+^	BHEH^+^	IHEH^+^
E_HOMO_ (eV)	− 5.805	− 5.877	− 5.990	− 3.658	− 3.897	− 3.540
E_LUMO_ (eV)	− 2.785	− 3.017	− 3.256	− 3.191	− 3.565	− 3.241
∆E_gap_ (eV)	3.019	2.859	2.734	0.467	0.333	0.299
I = − E_HOMO_ (eV)	5.805	5.877	5.990	3.658	3.897	3.540
A = − E_LUMO_ (eV)	2.785	3.017	3.256	3.191	3.565	3.241
χ = (I + A)/2 (eV)	4.295	4.447	4.623	3.425	3.731	3.391
η = (I − A)/2 (eV)	1.510	1.430	1.367	0.233	0.166	0.150
S = 1/η	0.6624	0.6995	0.7315	4.2834	6.0136	6.6847
ω = χ2/2η	6.110	6.916	7.817	25.117	41.859	38.425
nucleophilicity (ε)	0.164	0.145	0.128	0.040	0.024	0.026
ΔN = (χ_Fe_ − χ_inh_)/2(η_Fel_ + η_inh_)	0.1242	0.0780	0.0171	2.6674	2.8231	4.2761
ΔE = (χ_Fe_− χ_inh_)^2^/4(η_Fe_ + η_inh_)	0.023	0.009	0.0004	1.661	1.325	2.735
ω^+^= (I + 3 A)^2^/16(I − A)	4.151	4.872	5.676	23.434	40.014	36.749
ω^−^= (A + 3I)^2^/16(I − A)	8.446	9.319	10.300	26.858	43.745	40.139
Δω^±^ = ω^+^ +ω^−^	12.597	14.190	15.976	50.292	83.759	76.888
$\:\varDelta\:{E}_{Back-donation}=\:-\frac{\eta\:}{4}$	− 0.377	− 0.357	− 0.342	− 0.058	− 0.042	− 0.037
Binding energy (ev)	− 159.841	− 207.926	− 202.17	− 162.190	− 209.506	− 209.464
TE (ev)	− 31,822	− 36,994	− 37,429	− 31,838	− 37,009	− 37,475

**Fig. 17:**
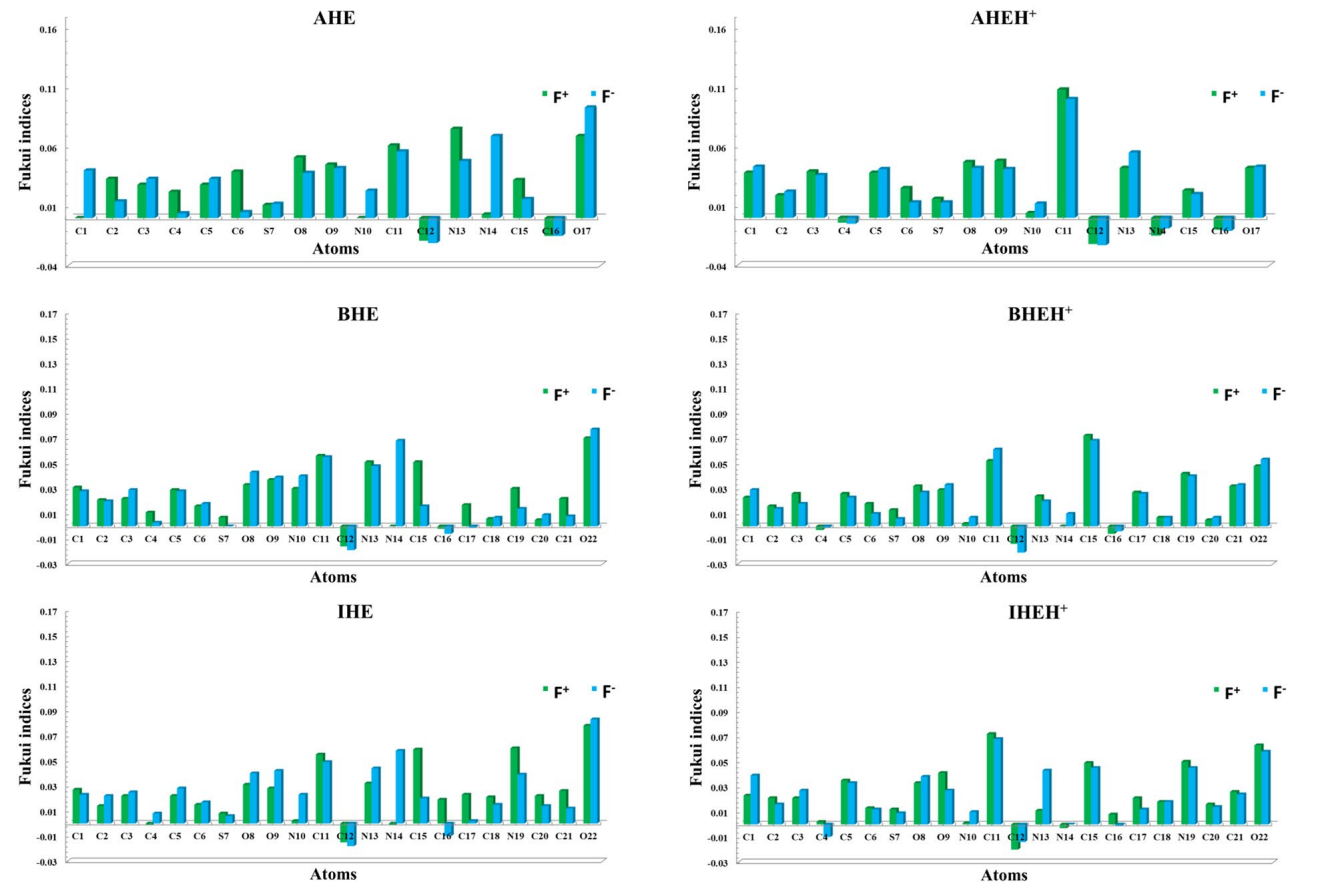
Fukui indices of neutral and protonated forms of the **AHE**,** BHE**,** and IHE** inhibitors.

**Fig. 18:**
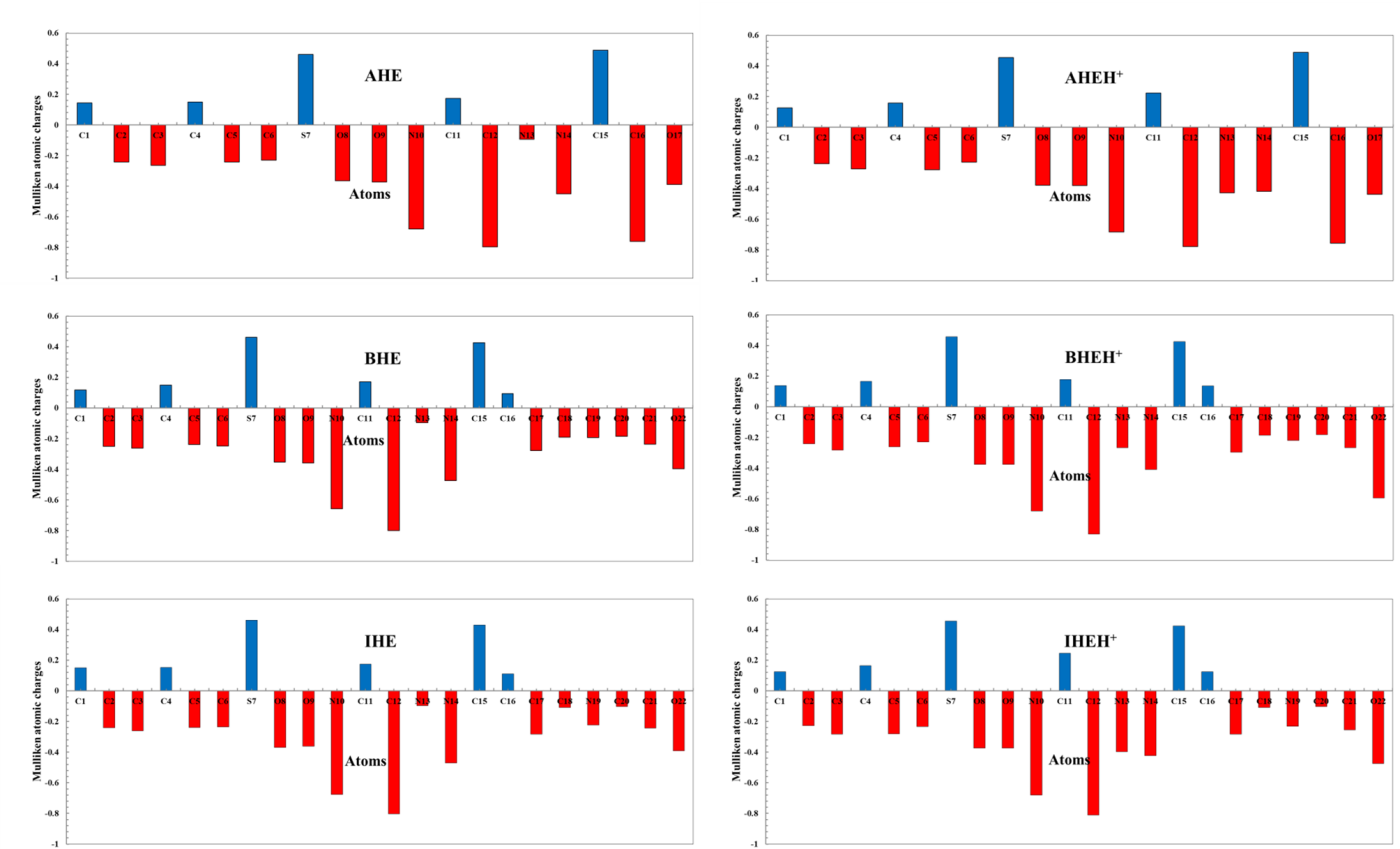
Mulliken atomic charge distribution of neutral and protonated forms of the **AHE**,** BHE**,** and IHE** inhibitors.

The Fukui indices for the studied neutral **AHE**, **BHE**, and **IHE** inhibitors are shown in Table S1. The highest values of $\:{f}_{k}^{+}$ and$\:\:\:{\varDelta\:f}_{k}>\:0$ identify electron-deficient centers, while the highest values of $\:{\:f}_{k}^{-\:}$ and $\:\:{\varDelta\:f}_{k}<$ 0 indicates sites susceptible to attack by electrophiles^70,83^.

According to the measured Fukui indices, the record favourable sites for nucleophilic attack in **AHE** are C2, C4, C6, O8, O9, C11, C12, N13, and C15; in **BHE**, they are C1, C2, C4, C5, S7, C11, C12, N13, C15, C16, C17, C19, and C21, and in **IHE**, they are C1, C11, C12, C15, C16, C17, C18, N19, C20, and C21. The most reactive positions toward electrophiles are C1, C3, C5, S7, N10, N14, and O17 in **AHE**; C3, C6, O8, O9, N10, N14, C18, C20, and O22 in **BHE**; and C2, C3, C4, C5, C6, O8, O9, N10, N13, N14, and O22 in **IHE**. Additionally, Table S1, Figs. 17 and 18 indicate that the **AHE**, **BHE**, and **IHE** molecules carry a negative charge around the carbon, oxygen, and nitrogen atoms, which have a significant impact on donating electrons to the CS surface. Finally, the investigation of electrophilic in addition to nucleophilic centers was matched to the frontier distribution of the **AHE**, **BHE**, and **IHE** inhibitor molecules, showing that the distributions of active sites designated with Fukui indices align well with the HOMO and LUMO electron densities^84^.

Table S2, Figs. 17 and 18 identifie the favored sites for nucleophilic and electrophilic attacks in **AHEH**^**+**^, **BHEH**^**+**^, **and IHEH**^**+.**^ After these molecules are protonated, there is a minor decrease in the magnitude of negative atomic charges, suggesting these sites could be potential points of interaction on the CS surface. This supports the idea that atoms in **AHE**, **BHE**, and **IHE** inhibitors are more likely to donate their electrons into the empty d-orbitals of the central CS metal atom^85^.

### Monte Carlo (MC) simulations

**Fig. 19:**
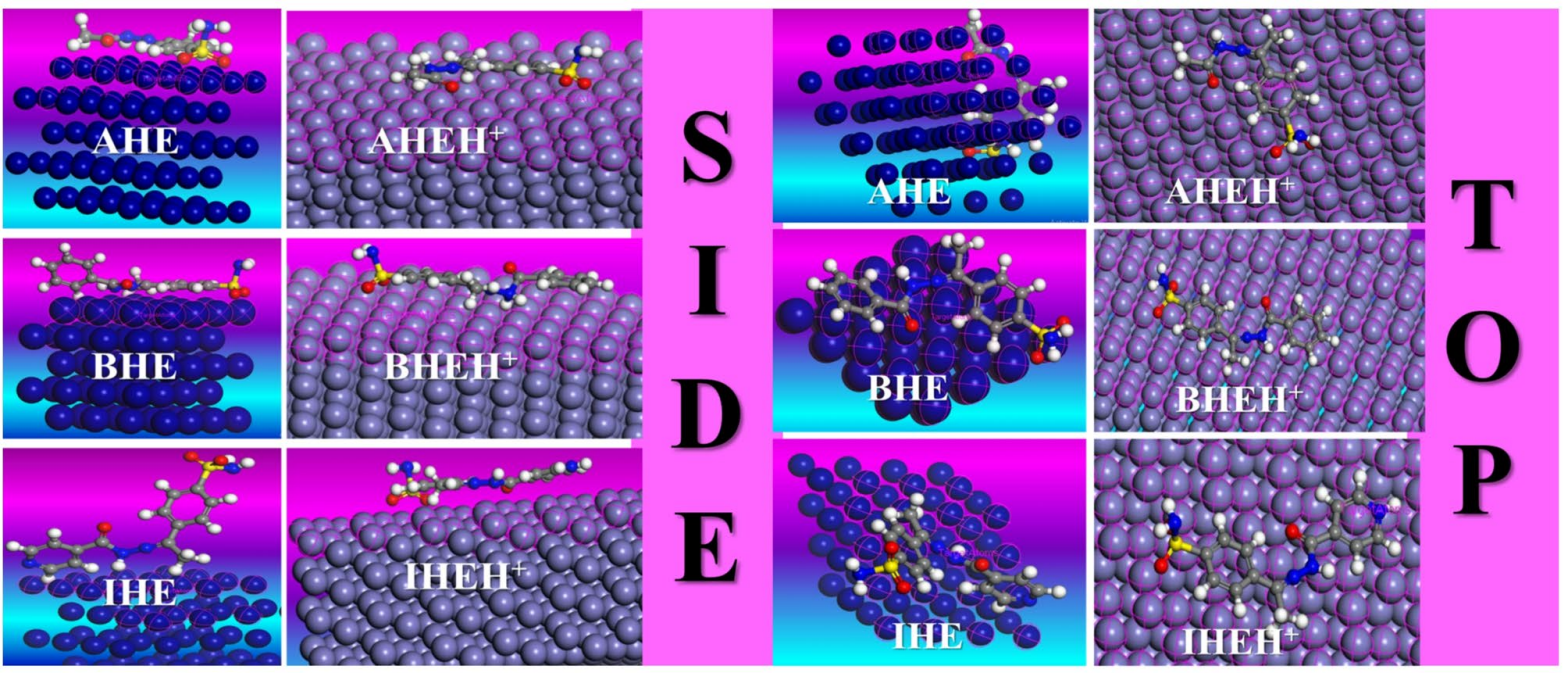
Top and side views of neutral and protonated forms of the **AHE**,** BHE**,** and IHE** inhibitors adsorption on the Fe (1 1 0) surface in gas phase.

**Fig. 20:**
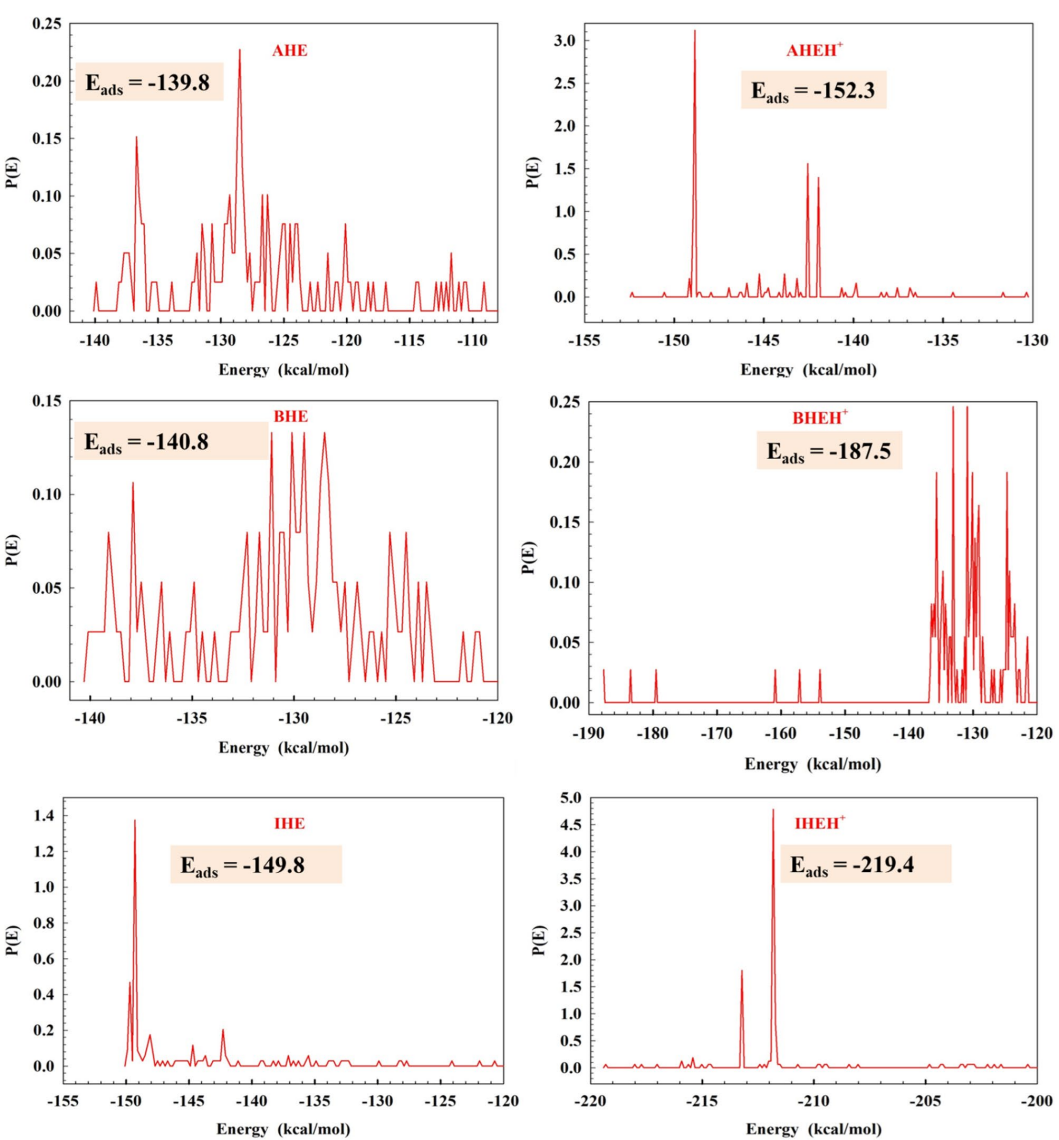
Adsorption energy distributions of neutral and protonated forms of the **AHE**,** BHE**,** and IHE** inhibitors on the Fe (1 1 0) surface in MD simulations at 298 K.

On the way for exploration of the interaction between prepared hydrazone derivatives and the CS surface, an MD simulation study was conducted. Using the adsorption locator module, the most stable adsorption arrangements of the inhibitors in the gas phase are presented in Fig. 19, which shows the inhibitors from top and side perspectives on the CS surface. MC output parameters are detailed in Table 14^86^. Figure 19 illustrates that the **AHE**, **BHE**, and **IHE** inhibitor molecules tend to adsorb in a direction parallel to the surface Fe (1 1 0), achieving the best possible coverage. This arrangement enables the active sites to interact more efficiently with the Fe (1 1 0) surface, leading to improved protective affinity. According to Table 14, **IHE** exhibits the highest adsorption energy throughout the simulation, representing it is the best efficient inhibitor. Moreover, the negative values of adsorption energies suggest the adsorption arrangement is established and spontaneous. The low adsorption energy $\:{\mathrm{E}}_{\mathrm{a}\mathrm{d}\mathrm{s}}$ values and high binding energy values (Eq. 17) imply that **AHE**, **BHE**, and **IHE** inhibitors will adsorb more effectively on the Fe (1 1 0) surface, ranking in effectiveness by way of **IHE** > **BHE** > **AHE** (Fig. 20).

17 $$\:{\mathrm{E}}_{\mathrm{b}\mathrm{i}\mathrm{n}\mathrm{d}}={-\mathrm{E}}_{\mathrm{a}\mathrm{d}\mathrm{s}\:}$$

The interaction energy ($\:{\mathrm{E}}_{\mathrm{i}\mathrm{n}\mathrm{t}\mathrm{e}\mathrm{r}\mathrm{a}\mathrm{c}\mathrm{t}\mathrm{i}\mathrm{o}\mathrm{n}}$) of the constructed system (Fig. 14) was calculated using Eq. (18)^80,87^.

18 $$\:{\mathrm{E}}_{\mathrm{i}\mathrm{n}\mathrm{t}\mathrm{e}\mathrm{r}\mathrm{a}\mathrm{c}\mathrm{t}\mathrm{i}\mathrm{o}\mathrm{n}}={\mathrm{E}}_{\mathrm{t}\mathrm{o}\mathrm{t}\mathrm{a}\mathrm{l}\:}-\left({\mathrm{E}}_{\mathrm{s}\mathrm{u}\mathrm{r}\mathrm{f}\mathrm{a}\mathrm{c}\mathrm{e}+{H}_{2}\mathrm{O}+{H}_{3}\mathrm{O}+{Cl}^{-}}+{\mathrm{E}}_{\mathrm{i}\mathrm{n}\mathrm{h}\mathrm{i}\mathrm{b}\mathrm{i}\mathrm{t}\mathrm{o}\mathrm{r}}\right)$$

**Fig. 21:**
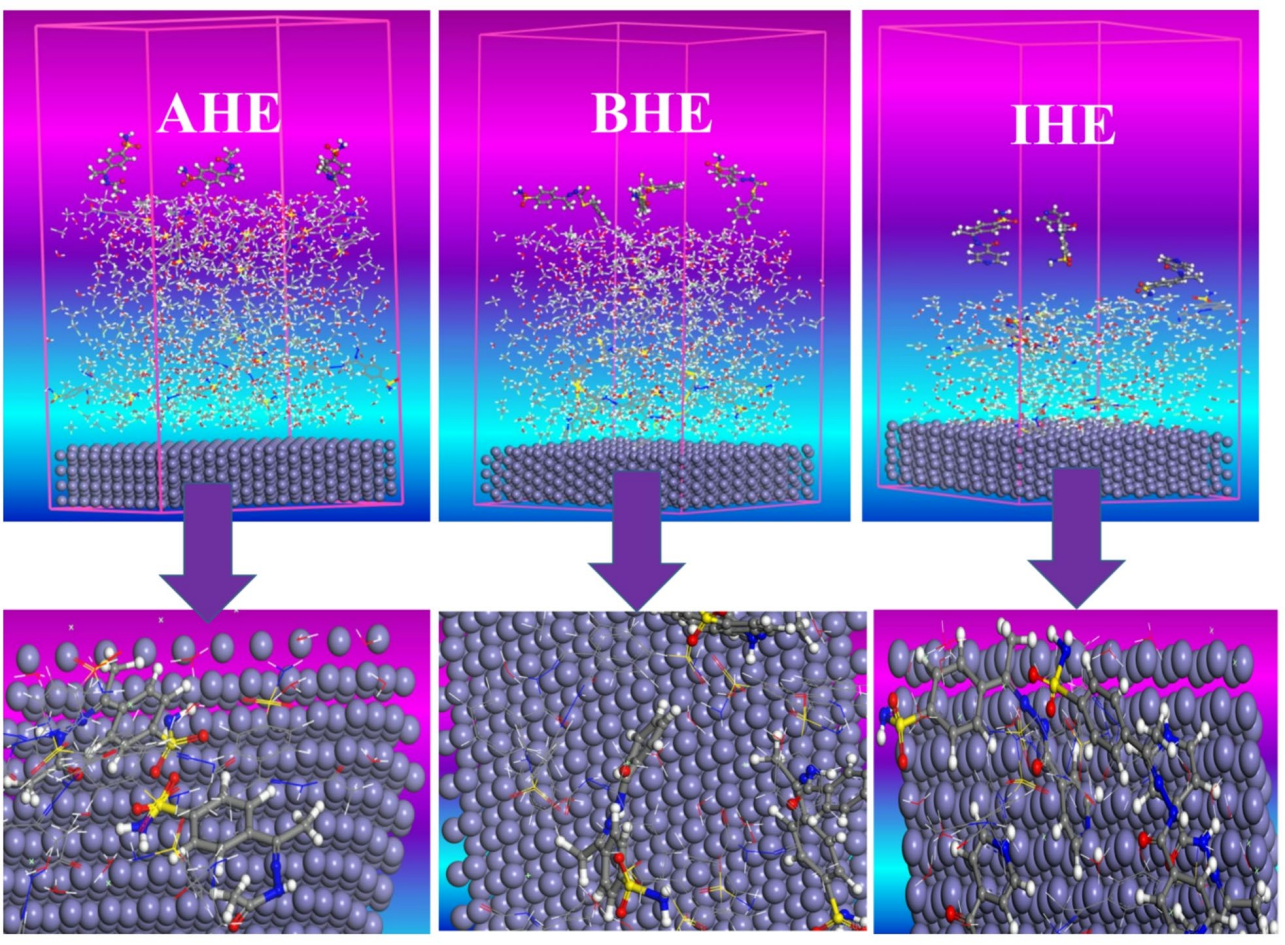
The Layer Builder of **AHE**,** BHE**,** and IHE** inhibitors on the Fe (1 1 0) surface in an amorphous cell module at 298 K.

Here, $\:{\mathrm{E}}_{\mathrm{t}\mathrm{o}\mathrm{t}\mathrm{a}\mathrm{l}\:}$ represents the overall energy of the all system; $\:{\mathrm{E}}_{\mathrm{s}\mathrm{u}\mathrm{r}\mathrm{f}\mathrm{a}\mathrm{c}\mathrm{e}+{H}_{2}\mathrm{O}+{H}_{3}\mathrm{O}+{Cl}^{-}}$ refers to the collective energy of the Fe (110) surface and the solution without the inhibitors, and $\:{\mathrm{E}}_{\mathrm{i}\mathrm{n}\mathrm{h}\mathrm{i}\mathrm{b}\mathrm{i}\mathrm{t}\mathrm{o}\mathrm{r}}$ is the energy of inhibitors. The values of energies are for **AHE** (E_total_ = 58042.97, E_surface + solution_ = 29615.58, E_inhibitor_= 28725.64, and **E**_**interaction**_= **-298.25**), **BHE** (E_total_ = 31316.41, E_surface + solution_ = 25491.71, E_inhibitor_= 6124.38 and **E**_**interaction**_= **-299.68**), and **IHE** (E_total_ = 52571.49, E_surface + solution_ = 29948.31, E_inhibitor_= 24248.36 and **E**_**interaction**_= **-1625.19**) kcal mol^[−1^, respectively, Fig. 21.

The bonding interactions between the hydrazone derivative molecules and the CS surface were analyzed using the radial distribution function (RDF) approach, as outlined in Eq. (19).

19 $$\:g_{{AB}} \left( r \right) = \frac{1}{{\left\langle {\rho _{B} } \right\rangle }} \times \frac{1}{{N_{A} }}\sum\limits_{{i \in A}}^{{N_{A} }} {\sum\limits_{{j \in B}}^{{N_{B} }} {\frac{{\delta (r_{{ij}} - r)}}{{4\pi r^{2} }}} }$$

**Fig. 22:**
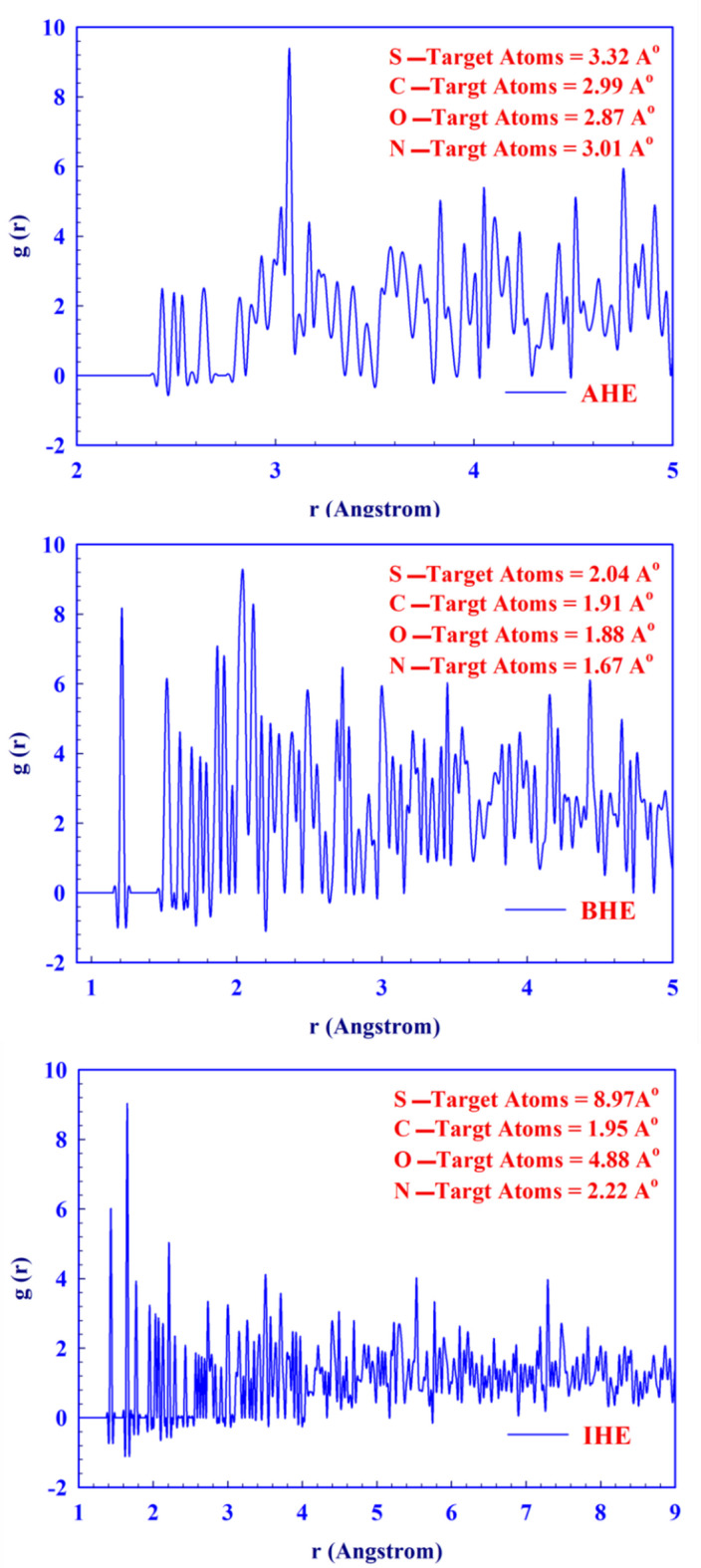
RDF of **AHE**,** BHE**,** and IHE** inhibitors on the Fe (1 1 0) surface in MD simulations at 298 K.

The RDF profiles (Fig. 22) of **AHE**, **BHE**, and **IHE** compounds reveal distinct atomic structuring around Fe (110) surface atoms. **AHE** shows moderate ordering with a main peak near 3.0 Å and limited long-range interactions, suggesting a compact or semi-amorphous structure. **BHE** exhibits sharper, shorter-distance peaks near 1.9 Å, indicating strong local interactions and high packing density. In contrast, **IHE** features broader peaks extending to 9.0 Å, reflecting multi-shell coordination and extended frameworks, indicative of enhanced long-range order and a more open structure compared to **AHE** and **BHE**. Ultimately, surface analysis, theoretical studies, and experimental data are consistent with the outcomes of MD and RDF analyses.

**Table 14:** Molecular dynamic simulation outputs and descriptors of the studied neutral and protonated forms of **AHE**,** BHE**,** and IHE** inhibitors on Fe (1 1 0) surface.

Molecule/descriptors	Total energy	Adsorption energy	Rigid adsorption energy	Deformation energy	Binding energy
AHE	− 208.233	− 139.828	− 145.372	5.544	139.828
BHE	− 246.832	− 140.818	− 143.833	3.014	140.818
IHE	− 191.853	− 149.844	− 149.679	− 0.1654	149.844
AHEH^+^	− 218.464	− 152.335	− 161.69	9.355	152.335
BHEH^+^	− 225.045	− 187.516	− 133.502	− 54.0141	187.516
IHEH^+^	− 351.055	− 219.422	− 226.984	7.5617	219.422

#### Adsorption and Inhibition mechanism

**Fig. 23:**
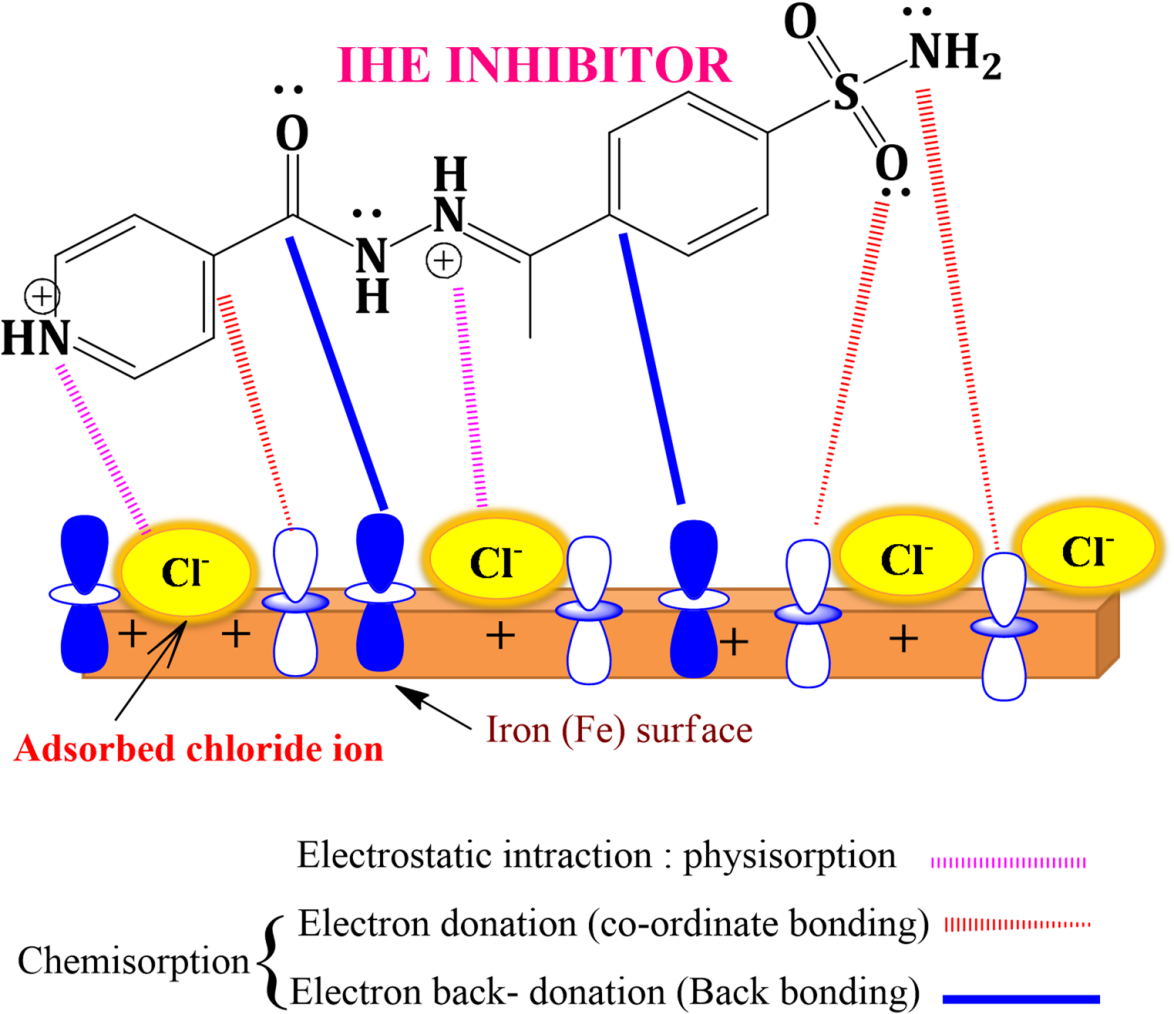
Schematic illustration of possible adsorption mechanisms of the **IHE** inhibitor on the carbon steel surface.

Hydrazone derivative inhibitor’s adsorption onto the CS surface is understood through the processes of physisorption, chemical adsorption, or a combination of both. Physisorption, where **AHE**, **BHE**, and **IHE** molecules can adsorb onto the CS surface through electrostatic interactions between the charged surface of Fe and the protonated **AHE**, **BHE**, and **IHE** molecules. Chemisorption occurs as a result of donor-acceptor interactions between lone electron pairs on heteroatoms, π-electrons from double bonds and aromatic rings, and the unoccupied d-orbitals of iron atoms. Calculated $\:{\varDelta\:G}_{ads}^{o}$values for **AHE**, **BHE**, and **IHE** suggest the hydrazone derivatives adsorption mechanism on the N80CS surface involves a combination of both physisorption and chemisorption. Therefore, **AHE**, **BHE**, and **IHE** inhibitors may adsorb either as cationic forms or as neutral molecules. For neutral molecules, adsorption likely occurs through chemisorption, where adsorbed water molecules are displaced from the steel surface, allowing heteroatoms to share electrons with Fe. Additionally, the adsorption of **AHE**, **BHE**, and **IHE** inhibitors onto the CS surface involves donor-acceptor interactions, where the π-electrons from the aromatic ring interact with the empty d-orbitals of the Fe metal atoms. When it comes to the protonated hydrazone derivatives adsorption, it was recognized that carbon steel surfaces in acidic solutions possess a positive charge^43^. This creates difficulty for the protonated inhibitor molecules to come near the surface because of the electrostatic repulsion between them. However, because chloride ions (Cl⁻) are less strongly hydrated, they can introduce extra –ve charges near the surface, facilitating the adsorption of +ve charges of **AHE**, **BHE**, and **IHE** molecules. The proposed mechanism of **IHE** induced suppression of CS is shown in Fig. 23.

## Conclusion

A QSAR model using the Genetic Function Approximation (GFA) approach was developed to predict the corrosion inhibition efficiency of three newly synthesized hydrazone derivatives. The predicted QSAR results show strong consistency with the experimental findings obtained through EIS and PDP. The activities of corrosion inhibition for **AHE**, **BHE**, and **IHE** against N80CS corrosion were evaluated using electrochemical techniques and theoretical approaches. As the concentrations of the **AHE**,** BHE**,** and IHE** inhibitors grew, so did their inhibitory effectiveness. Adsorption mechanisms of **AHE**, **BHE**, and **IHE** best fit Langmuir adsorption. PDP measurements indicated that the synthesized **AHE**, **BHE**, and **IHE** are inhibitors of mixed type. EIS analysis revealed that the inhibitors inhibited CS deterioration in 1.0 M HCl solutions, leading to an increase in charge transfer resistance as the concentrations of the inhibitor rose. A protective layer has developed on the CS surface because of the adsorption of **AHE**, **BHE**, and **IHE** molecules, as confirmed by AFM, SEM, EDX, and contact angle investigations. These films shield the carbon steel surface from being directly exposed to 1.0 M HCl solution. The results of the quantum computations showed that the effectiveness of **AHE**, **BHE**, and **IHE** in inhibiting is largely due to their abilities to donate and accept electrons. Overall, the findings as of both theoretical calculations and experimental studies align well, confirming the inhibition efficiencies of the synthesized **AHE**, **BHE**, and **IHE** inhibitors.

## Supplementary Information

Below is the link to the electronic supplementary material.

Supplementary Material 1

## Data Availability

The datasets used and/or analysed during the current study available from the corresponding author on reasonable request.
