# Supplementary material for: Theoretical–experimental study of new synthesized hydrazine–hydrazone benzenesulfonamide inhibitors for carbon steel in a 1.0 M HCI DFT, QSAR
Source: Sci Rep. 2026 May 29;16:17363. doi: 10.1038/s41598-026-39947-w (PMC13236985; doi:10.1038/s41598-026-39947-w)
Supplement: Supplementary file 1 — Supplementary Material 1 [file 41598_2026_39947_MOESM1_ESM.docx]

**Theoretical–Experimental Study of new synthesized Hydrazine–Hydrazone Benzenesulfonamide Inhibitors for Carbon Steel in a 1.0 M HCl: DFT, QSAR**

Walid E. Elgammal**^a^***, Reema H. Aldahiri**^b^**, Shereen M. Al-Shomar^c^, Saber M. Hassan^a^, Amr Gangan**^a^**, N.S. Abdelshafi**^d^***

^a^Chemistry Department, Faculty of Science (Boys), Al-Azhar University, Nasr City, 11884, Cairo, Egypt.

^b^Department of Chemistry, College of Science, University of Jeddah, Jeddah 21959, Saudi Arabia.

^c^Department of Physics, College of Science, University of Ha’il, P.O. Box 2440, Ha’il, Saudi Arabia

^d^Chemistry Department, Faculty of Education, Ain Shams University, Roxy, 11711, Cairo, Egypt.

**Corresponding authors:**

*** N.S.Abdelshafi**

**Tel.:** +201095670838, **E-mail:** nashwasaad@edu.asu.edu.eg ORCID: 0000-0001-5170-1745

Chemistry Department, Faculty of Education, Ain Shams University, Roxy, 11711, Cairo, Egypt.

* **Walid E. Elgammal**

Chemistry Department, Faculty of Science, Al-Azhar University, Nasr City, Cairo, Egypt, Tel: +201025267111, E-mail: [walidebaied.sci85@azhar.edu.eg](mailto:walidebaied.sci85@azhar.edu.eg), ORCID: 0000-0002-5982-5635, Al-Azhar University, El-Nasr Road, Nasr City, 11884, Cairo, Egypt.

**Supporting information**

**Supplementary Methods**

**Resources and Procedures:**

**Substances**

Commercial vendors, such as Sigma-Aldrich (USA), Alpha Chem (Pakistan), Fluka (USA), and Loba (India) supplied the solvents and preparative reagents. No additional purification was necessary for these chemicals. A common technique for monitoring reaction progress and determining product purity was the use of the systematic separation method (TLC) based on a silica gel sheet with an ultraviolet index, using methanol and dichloromethane in the percentage of 5: 95. An automatic system was used to measure the fusion points (Fp. °C) of crystalline samples held within capillary tubes using a 120/230V SMP50 Computerized Fusion Point App (Bibby Technical, Staffordshire, UK). To document infrared spectra, the potassium bromide disc method was used on the Nicolet iS10FT IR Spectrometer, Thermo Fisher Scientific Resolution 16 (cm^−1^). The JNM-ECA 500 II Made by the JEOL-JAPAN instrument was used to record 1H/13C spectra at 400 and 125 MHz, respectively, through a deuterated DMSO-d6 solution using the JEOL-JAPAN instrument. An organosilicon molecule, such as tetramethylsilane (TMS) having the formula Si(CH_3_)_4_, was employed as an internal reference. Coupling constants (J) are measured in hertz (Hz), and chemical changes are expressed in parts per million (ppm). Terms s, d, t, dd, and m, in that order, stand for singlet, doublet, triplet, doublet of doublet, and multiple. Additionally, "b s" denotes a broad singlet. The internal references used in ^13^C NMR were 39.9 ppm, while in ^1^H NMR they were the residual protons at 2.50 and 3.31 ppm. The inhibitors' mass spectrometry (MS) is measured analytically at the Regional Center for Mycology and Biotechnology (RCMB) of Al-Azhar University in Naser City, Cairo, Egypt, using a Thermo Scientific GCMS model (Isq Lt) and Thermo X-Calibur software (Shimadzu, Kyoto, Japan). The results of elemental analysis (C-H-N) were accurate to within 0.4% at the Regional Center for Microbiology and Biotechnology, Al-Azhar University, Cairo, Egypt.

Carbon steel sample was sourced from Central Metallurgical Research Institute in Egypt and fashioned into disc-shaped specimens with a 10 mm diameter. These specimens were polished using silicon carbide abrasive papers of progressively finer grits (60, 80, 120, 400, 800, 1000, in addition to 2000) to ensure a smooth surface and then cleaned with acetone before air-drying at ambient temperature.

**Electrochemical and Gravimetric weight loss measurements**

Electrochemical experiments were measured by the AC impedance technique (EIS). These experiments utilized a Galvanostat/potentiostat/ZRA analyzer (GAMRY Reference 3000) with a traditional configuration featuring mercury/mercurous chloride (Hg/Hg₂Cl₂) reference electrode in a saturated KCl solution and platinum wire mesh serving as the secondary electrode. The active area of the working electrode was set to 1.0 cm². In case of potentiodynamic polarization investigations, the potentials were varied from -0.3 to 1.0 mV by a scan rate for each experiment of 2.0 mV/s. Electrochemical impedance spectroscopy (EIS) was achieved employing a 10 mV signal strength sinusoidal oscillating electrical signal, across a frequency spectrum ranging from 100 kilohertz to 10 millihertz, with respect to open-circuit potential (OCP). The impedance data were evaluated using means of Gamry Echem Analyst software to select appropriate electrochemical equivalent circuits and determine EIS parameters. 1.0 M hydrochloric acid solutions prepared using concentrated HCl at 37% purity are used for all measurements of cleaned and dried N80 CS.

N80 carbon steel coupons (7 cm × 2 cm × 1 mm) were precisely weighed and then submerged in 100 mL of a 1.0 M hydrochloric acid (HCl) solution, under conditions including and excluding a range of **AHE**, **BHE**, and **IHE** inhibitor concentrations. Corrosion tests were conducted over a 24-hour period at temperatures varying between 298 K and 358 K. After immersion, the coupons were flushed with distilled water, cleaned ultrasonically using acetone, and ethanol. Afterward, they were dried and weighed again. All tests were performed three times to ensure accuracy and reproducibility, with mean weight loss calculated.

**N80 carbon steel surface examination**

The confirmation of **AHE, BHE, and IHE** inhibitors adsorption was established through the surface characterization of N80 CS specimens measuring$2\times2.50\times0.04 cm$. This characterization was conducted both before the inhibition process and after via immersing the testers in 1.0 M corrosive solutions, both with besides without the existence of 400 ppm inhibitors, for a duration of one day at 298 K. Upon completion of the test stage, testers are pulled out from the solutions, rinsed by way of bi-distilled water, subjected to a 5-minute ultrasonic bath with acetone for cleaning and subsequently left to dry at 298K. The surfaces were analyzed by SEM, the scanning electron microscope, Prisma E (Thermo Fisher Company), an accelerating voltage of 30 kv, an LFD detector, a 3.5 spot size, and operating in low vacuum mode at 250× magnification. (EDX) characterization, Energy dispersive X-ray analysis was employed by means of the Thermo Scientific Dry/Wet system and Scanning probe technique using atomic-scale forces (AFM) was carried out with Flex Axiom Nanosurf C3000. Additionally, the angle of static contact measurements were taken via a KRUSS DSA2S8 device, operating at 12 VDC and 40 W.

**Theoretical analysis**

MD is employed for studying the behavior of **AHE, BHE, and IHE** inhibitors adsorption on a CS surface. Condensed-phase Optimised Molecular Potentials for Atomistic Simulation Studies, or COMPASS, the force field was used to simulate these molecules' interactions on the Fe(110) surface. Dimensions of the simulation box were 24.320 Å × 24.320 Å × 39.100 Å. [1].

**Synthesis and characterization of predicted inhibitors**

**A comprehensive method for the synthesis of intermediate 2.**


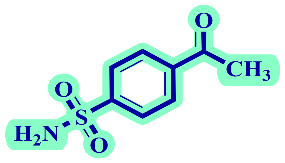
 In a two hundred fifty milliliter conical flask fitted with an automated stirrer, 0.1mmol of crude sulfonyl chloride derivative 1 was liquefied in 25 mL of DCM in the comportment of a catalytic amount (3 mL) of a mild base such as pyridine. Stir the mixture for 30 minutes at 20 °C. Then, slowly add 50 mL of a solution of 25% NH_3_ in H_2_O. During the addition, a thick white precipitate will form. Once the addition is complete, stir the mixture for another 3 hours. After that, concentrated hydrochloric acid was employed to acidify the reaction mixture to a pH of 2. Through the utilization of filtration, the solid material was separated and subsequently rinsed with chill water to ensure comprehensive purification. Subsequently, the solid was subjected to vacuum drying, resulting in the formation of off-white crystals. The crystals, which served as the representation of the title compound, demonstrated a give of 76% and a fusion point of 181 °C.

**General Synthetic Procedure for Compounds IHE, BHE, and AHE.**

A solution of intermediate **2** (0.1 mmol) in 20 ml of anhydrous C_2_H_5_OH with an analytical volume of anhydrous acetic acid was added to the applicable hydrazide 3-5 (0.1 mmol). Next, the reaction mixture was refluxed for three to four hours. After confirming the reaction's completion with TLC analysis, the white solid precipitate produced was filtered using Whatman filter paper. The solid was then rinsed three times with hot C_2_H_5_OH and dried in the air, to obtain pure products, and recrystallization from dioxane was performed.

**(E)-4-(1-(2-isonicotinoylhydrazineylidene)ethyl)benzenesulfonamide (IHE)**

**
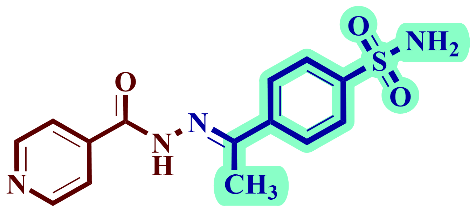
**Off-white-powdered amorphous, 96% Yield, Melting point: 276- 278 °C; IR (KBr, ⱱ cm^−1^): 3329, 3239cm^-1^ (NH, & NH_2_), 3041cm^-1^ (aromatic-CH), 2937cm^-1^ (aliphatic-CH), 1687cm^-1^ (C=O), 1605cm^-1^ (C=N), and 1321, 1153cm^-1^ (SO_2_). ^1^H NMR (400 MHz, DMSO-*d*_6_) δ_H_= 11.12 (s, 1H, NH, commutable by D_2_O), 8.78 (dd, *J* = 5.1 Hz, 2H, AB-Ar-H), 8.05 (dd, *J* = 8.1 Hz, 2H, AB-Ar-H), 7.90 (dd, *J* = 8.1 Hz, 2H, AB-Ar-H), 7.82 (dd, *J* = 4.9 Hz, 2H, AB-Ar-H), 7.45 (s, 2H, NH_2_, commutable by D_2_O), 2.47 (s, 3H, CH_3_).^13^C NMR (101 MHz, DMSO) δ_C_= 163.31, 155.49, 150.59, 145.18, 141.37, 129.32, 127.52, 126.22, 122.42, 15.29. EI-MS (m/z) **=**318 [%]: [M^+^, (21.72%)], Anal. Calcd for C_14_H_14_N_4_O_3_S (318.35): C, 52.82; H, 4.43; N, 17.60; Found: C, 52.94; H, 4.54; N, 17.73%.

**
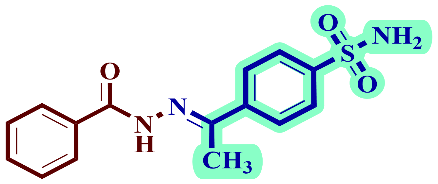
2.5.4. (E)-4-(1-(2-benzoylhydrazineylidene)ethyl)benzenesulfonamide (BHE)**

White-powdered amorphous, 87% Yield, Melting point: 270- 271 °C; IR (KBr, ⱱ cm^−1^): 3318 cm^-1^ (NH), 3287, 3196m^-1^ (NH_2_), 3090cm^-1^ (aromatic-CH), 2803cm^-1^ (aliphatic-CH), 1651cm^-1^ (C=O), 1602cm^-1^ (C=N), and 1342, 1165cm^-1^ (SO_2_). ^1^H NMR (400 MHz, DMSO-*d*_6_) δ_H_= 10.89 (s, 1H, NH, commutable by D_2_O), 8.03 (s, 1H, Ar-H), 7.88 (d, *J* = 8.8 Hz, 4H, Ar-H), 7.60 (t, *J* = 7.3 Hz, 1H, Ar-H), 7.53 (t, *J* = 7.4 Hz, 2H, Ar-H), 7.44 (s, 2H, NH_2_, commutable by D_2_O), 2.42 (s, 3H, CH_3_). ^13^C NMR (101 MHz, DMSO) δ_C_= 164.75, 144.89, 141.65, 134.36, 132.10, 129.32, 128.80, 127.56, 127.33, 126.20, 14.98. EI-MS (m/z) **=**317 [%]: [M^+^, (25.03%)], Anal. Calcd for C_15_H_15_N_3_O_3_S (317.36): C, 56.77; H, 4.76; N, 13.24; Found: C, 56.89; H, 4.87; N, 13.35%.

**
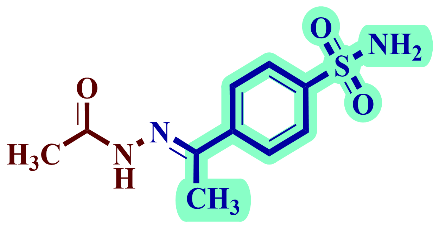
1.5.5. (E)-4-(1-(2-acetylhydrazineylidene)ethyl)benzenesulfonamide (AHE)**

Pale-yellow powdered amorphous, 80% Yield, Melting point: 219 - 220 °C; IR (KBr, ⱱ cm^−1^): 3539 cm^-1^ (NH), 3273, 3227m^-1^ (NH_2_), 3017cm^-1^ (aromatic-CH), 1669cm^-1^ (C=O), 1613cm^-1^ (C=N), and 1329, 1163cm^-1^ (SO_2_). ^1^H NMR (400 MHz, DMSO-*d*_6_) δ_H_= 10.58 (s, 1H, NH, commutable by D_2_O), 7.94 (dd, *J* = 7.8 Hz, 2H, AB-Ar-H), 7.84 (dd, *J* = 8.6 Hz, 2H, AB-Ar-H), 7.41 (s, 2H, NH_2_, commutable by D_2_O), 2.27 (s, 3H, CH_3_), 2.26 (s, 3H, CH_3_). ^13^C NMR (101 MHz, DMSO) δ_C_ = 173.58, 146.01, 144.45, 129.31, 126.84, 126.18, 21.37, 13.99. EI-MS (m/z) **=** 255 [%]: [M^+^, (26.14%)], Anal. Calcd for C_10_H_13_N_3_O_3_S (255.29): C, 47.05; H, 5.13; N, 16.46; Found: C, 47.16; H, 5.25; N, 16.59%.

**Supplementary results.**

**Design a QSAR Model using GFA method.**

GFA method is applied in statistical analysis to investigate and clarify QSAR model. This process includes performing a univariate analysis of inhibition (refer to Table 1 and Fig. 6a), constructing a study table (Table 2) that compiles all relevant physicochemical parameters alongside the experimental inhibition efficiency for 10 hydrazone derivative inhibitors, generating a correlation matrix, determining regression parameters (Table 3), validating the GFA model (Table 4), and deriving equations (Table 5) used to predict the inhibition efficiency for **AHE, BHE** and **IHE** (Table 6).

Table 1 shows the outcomes of a univariate analysis of inhibition, presenting various statistical metrics such as standard deviation, mean absolute deviation, variance, Skewness, and kurtosis. Skewness measures the asymmetry of the data, where negative values suggest a left-skewed distribution. Kurtosis evaluates how the data distribution compares to a normal distribution, with 0 representing normality; positive kurtosis indicates a more pronounced peak, while negative values suggest a flatter distribution. These metrics help assess the data's accuracy and normality for additional statistical evaluation [2].

Table 3 presents a correlation matrix displaying the relationship coefficients between different columns. A value of +1.0 or -1.0 indicates a strong relationship, while values closer to 0.0 suggest a weak or no relationship. The diagonal values are always 1.0. To enhance clarity, the cells are color-coded: orange represents values between +0.9 and +1.0 or -0.9 and -1.0, yellow for values between +0.7 and +0.9 or -0.7 and -0.9, and white for values falling between -0.7 and +0.7 [3].

The correlation matrix is used in regression analysis with GFA (Genetic Function Approximation) to compare descriptor variables with the calculated inhibition values. Important considerations include preventing overfitting by maintaining an appropriate ratio of measured values to descriptor variables, and aiming for a simplified equation that aligns with theoretical understanding.[4, 5].

To assess the fitness of a GFA model during the evolutionary process, various statistical measures can be utilized. The Friedman test for lack of fit (LOF) is often favored over the conventional least squares error metric. In Materials Studio [6], a revised version of Friedman’s initial equation [7] ] is applied to determine the LOF, as demonstrated in Eq. (1) [8]

$LOF= \frac{SSE}{{(1-\frac{c+dp}{M})}^{2}}$ (1)

Table 4 outlines the input parameters used for predicting the GFA analysis and specifies whether the GFA algorithm successfully converged within a predefined number of generations. Convergence occurs when the population score remains stable for an extended period, whether it is the highest performing model's score or the average score across all models. Once this happens, subsequent generations are disregarded [9].


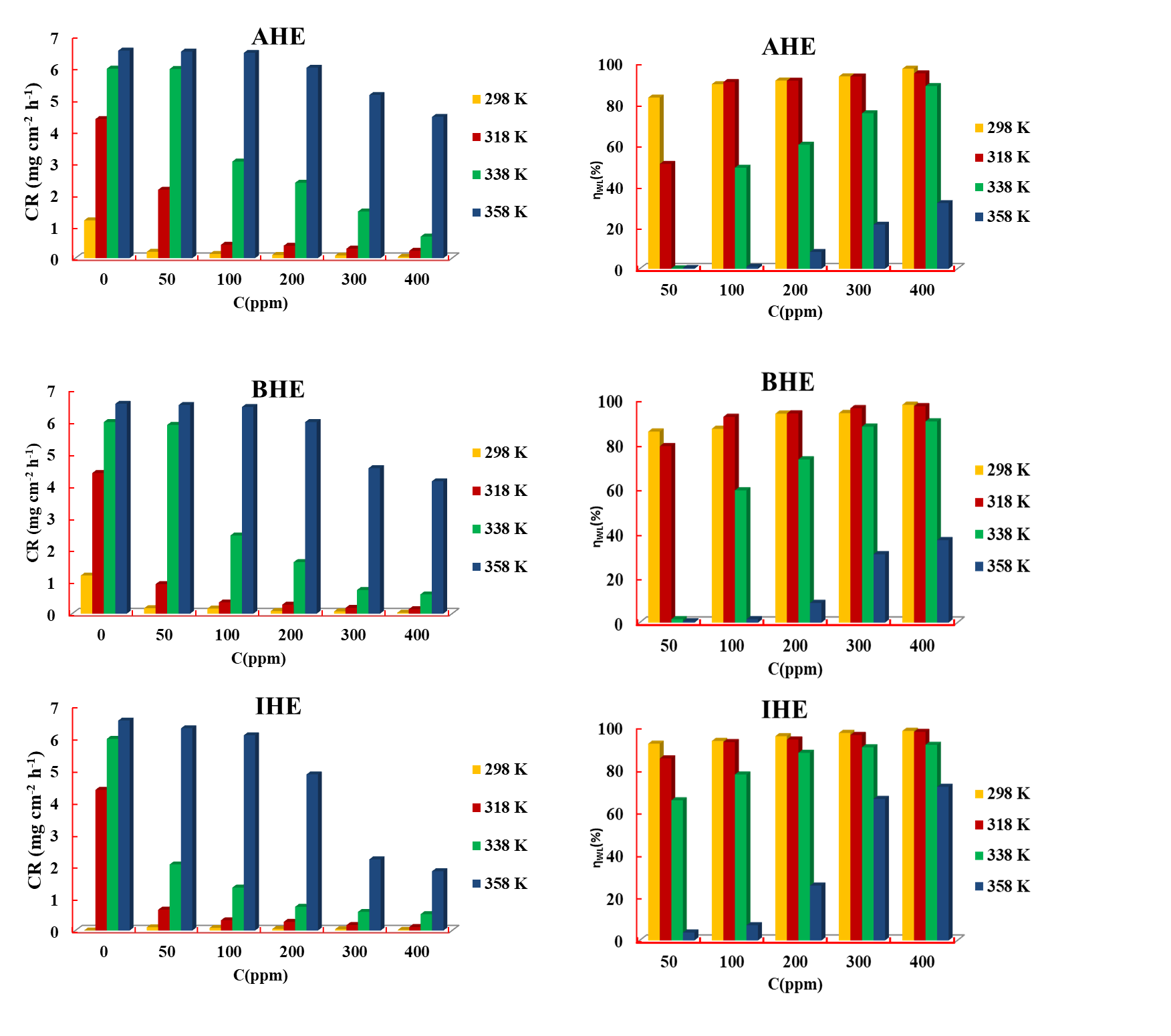


**Fig. S1.** Variation of corrosion rate and inhibition efficiency with concentrations of **AHE**, **BHE**, and **IHE** inhibitors by weight loss study at different temperature.

**
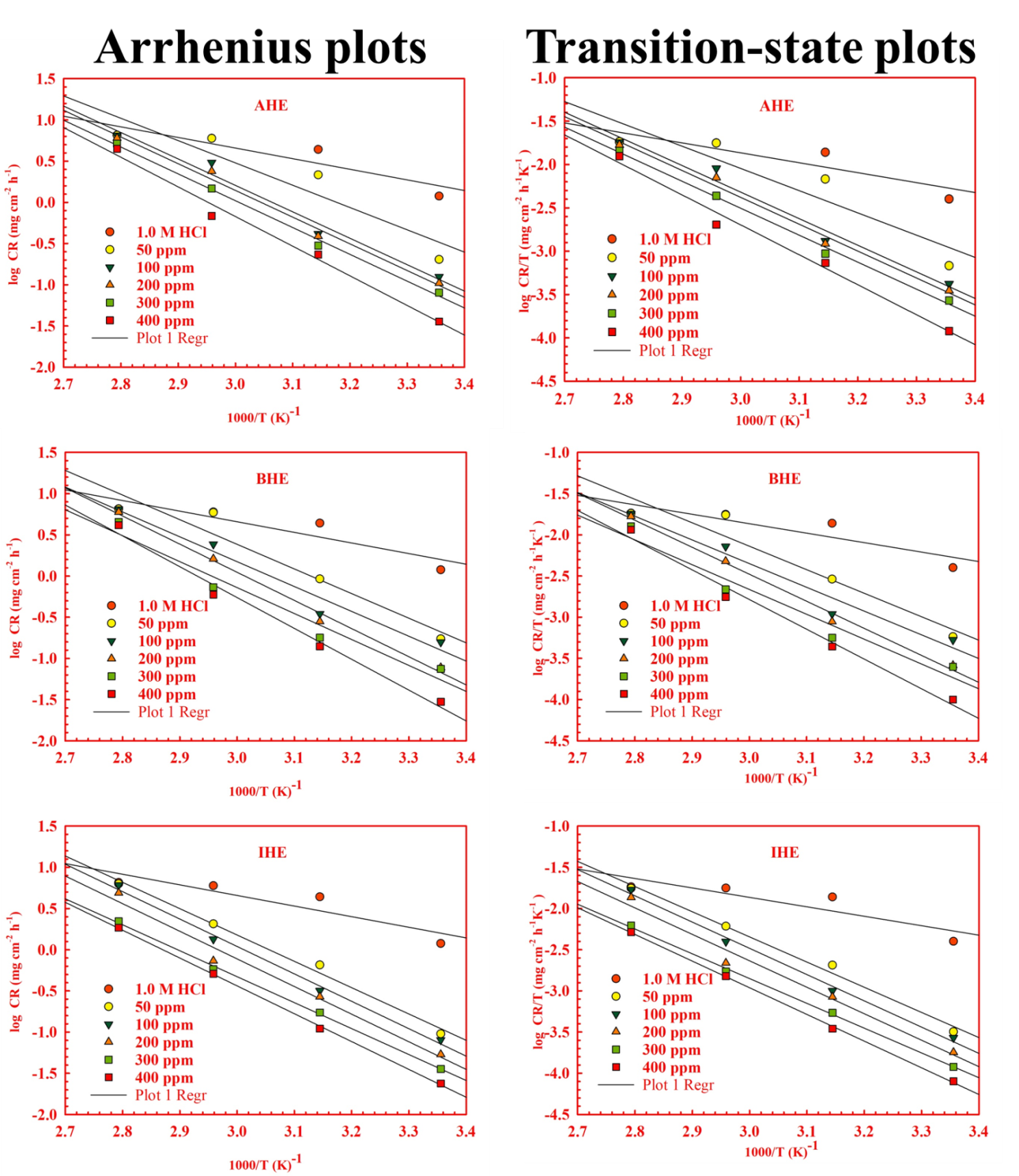
 Fig. S2.**Arrhenius and transition-state plots for carbon steel in 1.0 M HCl solution in the absence and presence of different concentrations of **AHE**, **BHE**, and **IHE** inhibitors by weight loss study.


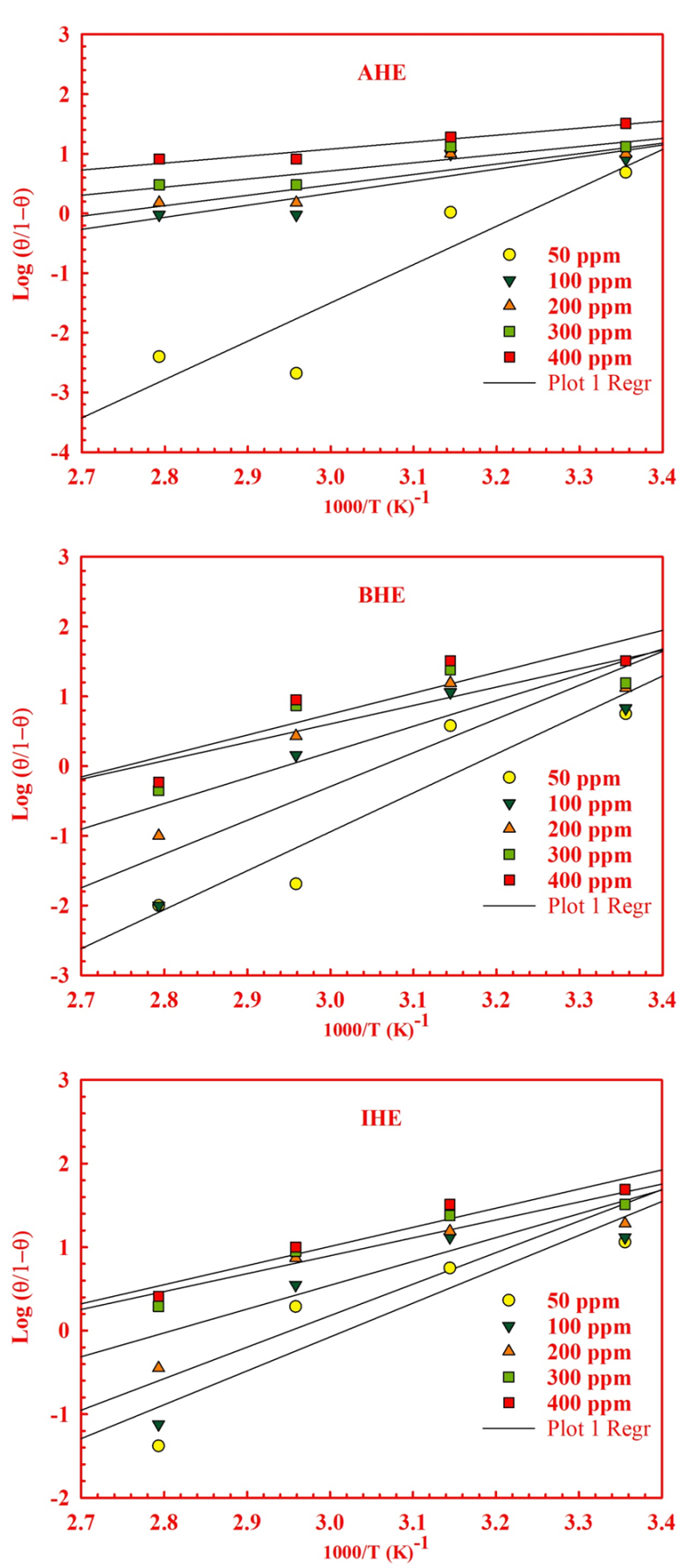


Fig. S3. Plot of $log (\theta/1-\theta)$ against 1000/T for carbon steel in 1.0 M HCl solutions in the absence and presence of different concentrations of **AHE**, **BHE**, and **IHE** inhibitors by weight loss study at different temperature.

Table S1

Mullikan charges and Fukui index values for studied neutral AHE, BHE, and IHE inhibitors.

| **AHE** | | | | | **BHE** | | | | | **IHE** | | | | |
| --- | --- | --- | --- | --- | --- | --- | --- | --- | --- | --- | --- | --- | --- | --- |
| **Atoms** | **Mulliken atomic charges** | $\mathbf{f}_{\mathbf{k}}^{\mathbf{-}}$ | $\mathbf{f}_{\mathbf{k}}^{\mathbf{+}}$ | $\boldsymbol{\Delta}\mathbf{f}_{\mathbf{k}}$ | **Atoms** | **Mulliken atomic charges** | $\mathbf{f}_{\mathbf{k}}^{\mathbf{-}}$ | $\mathbf{f}_{\mathbf{k}}^{\mathbf{+}}$ | $\boldsymbol{\Delta}\mathbf{f}_{\mathbf{k}}$ | **Atoms** | **Mulliken atomic charges** | $\mathbf{f}_{\mathbf{k}}^{\mathbf{-}}$ | $\mathbf{f}_{\mathbf{k}}^{\mathbf{+}}$ | $\boldsymbol{\Delta}\mathbf{f}_{\mathbf{k}}$ |
| **C1** | 0.143 | 0.040 | 0.029 | -0.011 | **C1** | 0.118 | 0.028 | 0.031 | 0.003 | **C1** | 0.151 | 0.023 | 0.027 | 0.004 |
| **C2** | -0.243 | 0.014 | 0.033 | 0.019 | **C2** | -0.251 | 0.020 | 0.021 | 0.001 | **C2** | -0.241 | 0.022 | 0.014 | -0.008 |
| **C3** | -0.264 | 0.033 | 0.028 | -0.005 | **C3** | -0.262 | 0.029 | 0.022 | -0.007 | **C3** | -0.262 | 0.025 | 0.022 | -0.003 |
| **C4** | 0.150 | 0.004 | 0.022 | 0.018 | **C4** | 0.149 | 0.003 | 0.011 | 0.008 | **C4** | 0.152 | 0.008 | -0.001 | -0.009 |
| **C5** | -0.243 | 0.033 | 0.028 | -0.005 | **C5** | -0.239 | 0.028 | 0.029 | 0.001 | **C5** | -0.240 | 0.028 | 0.022 | -0.006 |
| **C6** | -0.231 | 0.005 | 0.039 | 0.034 | **C6** | -0.248 | 0.018 | 0.016 | -0.002 | **C6** | -0.236 | 0.017 | 0.015 | -0.002 |
| **S7** | 0.460 | 0.012 | 0.011 | -0.001 | **S7** | 0.461 | 0.000 | 0.007 | 0.007 | **S7** | 0.461 | 0.006 | 0.008 | 0.002 |
| **O8** | -0.365 | 0.038 | 0.051 | 0.013 | **O8** | -0.354 | 0.043 | 0.033 | -0.01 | **O8** | -0.370 | 0.040 | 0.031 | -0.009 |
| **O9** | -0.372 | 0.042 | 0.045 | 0.003 | **O9** | -0.360 | 0.039 | 0.037 | -0.002 | **O9** | -0.363 | 0.042 | 0.028 | -0.014 |
| **N10** | -0.680 | 0.023 | 0.000 | -0.023 | **N10** | -0.658 | 0.040 | 0.030 | -0.01 | **N10** | -0.677 | 0.023 | 0.002 | -0.021 |
| **C11** | 0.172 | 0.056 | 0.061 | 0.005 | **C11** | 0.170 | 0.055 | 0.056 | 0.001 | **C11** | 0.173 | 0.049 | 0.055 | 0.006 |
| **C12** | -0.797 | -0.021 | -0.019 | 0.002 | **C12** | -0.801 | -0.019 | -0.016 | 0.003 | **C12** | -0.803 | -0.018 | -0.015 | 0.003 |
| **N13** | -0.095 | 0.048 | 0.075 | 0.027 | **N13** | -0.097 | 0.048 | 0.051 | 0.003 | **N13** | -0.098 | 0.044 | 0.032 | -0.012 |
| **N14** | -0.450 | 0.069 | 0.003 | -0.066 | **N14** | -0.473 | 0.068 | 0.000 | -0.068 | **N14** | -0.471 | 0.058 | -0.001 | -0.059 |
| **C15** | 0.488 | 0.016 | 0.032 | 0.016 | **C15** | 0.426 | 0.016 | 0.051 | 0.035 | **C15** | 0.429 | 0.020 | 0.059 | 0.039 |
| **C16** | -0.760 | -0.015 | -0.015 | 0.000 | **C16** | 0.093 | -0.006 | -0.002 | 0.004 | **C16** | 0.111 | -0.010 | 0.019 | 0.029 |
| **O17** | -0.389 | 0.093 | 0.069 | -0.024 | **C17** | -0.277 | -0.001 | 0.017 | 0.018 | **C17** | -0.283 | 0.002 | 0.023 | 0.021 |
|  |  |  |  |  | **C18** | -0.191 | 0.007 | 0.006 | -0.001 | **C18** | -0.109 | 0.015 | 0.021 | 0.006 |
|  |  |  |  |  | **C19** | -0.192 | 0.014 | 0.030 | 0.016 | **N19** | -0.223 | 0.039 | 0.060 | 0.021 |
|  |  |  |  |  | **C20** | -0.186 | 0.009 | 0.005 | -0.004 | **C20** | -0.103 | 0.014 | 0.022 | 0.008 |
|  |  |  |  |  | **C21** | -0.237 | 0.008 | 0.022 | 0.014 | **C21** | -0.243 | 0.012 | 0.026 | 0.014 |
|  |  |  |  |  | **O22** | -0.397 | 0.077 | 0.070 | -0.007 | **O22** | -0.392 | 0.083 | 0.078 | -0.005 |

**Table S2**

Mullikan charges and Fukui index values for studied protonated **AHE, BHE, and IHE** inhibitors.

| **AHEH^+^** | | | | | **BHEH^+^** | | | | | **IHEH^+^** | | | | |
| --- | --- | --- | --- | --- | --- | --- | --- | --- | --- | --- | --- | --- | --- | --- |
| Atoms | Mulliken atomic charges | $f_{k}^{-}$ | $f_{k}^{+}$ | $\Delta f_{k}$ | Atoms | Mulliken atomic charges | $f_{k}^{-}$ | $f_{k}^{+}$ | $\Delta f_{k}$ | Atoms | Mulliken atomic charges | $f_{k}^{-}$ | $f_{k}^{+}$ | $\Delta f_{k}$ |
| **C1** | 0.126 | 0.043 | 0.038 | -0.005 | **C1** | 0.138 | 0.029 | 0.023 | -0.006 | **C1** | 0.124 | 0.039 | 0.023 | -0.016 |
| **C2** | -0.239 | 0.022 | 0.019 | -0.003 | **C2** | -0.241 | 0.014 | 0.016 | 0.002 | **C2** | -0.228 | 0.016 | 0.021 | 0.005 |
| **C3** | -0.273 | 0.036 | 0.039 | 0.003 | **C3** | -0.283 | 0.018 | 0.026 | 0.008 | **C3** | -0.283 | 0.027 | 0.021 | -0.006 |
| **C4** | 0.157 | -0.005 | -0.004 | 0.001 | **C4** | 0.165 | -0.001 | -0.003 | -0.002 | **C4** | 0.164 | -0.010 | 0.002 | 0.012 |
| **C5** | -0.278 | 0.041 | 0.038 | -0.003 | **C5** | -0.261 | 0.023 | 0.026 | 0.003 | **C5** | -0.280 | 0.033 | 0.035 | 0.002 |
| **C6** | -0.228 | 0.013 | 0.025 | 0.012 | **C6** | -0.230 | 0.010 | 0.018 | 0.008 | **C6** | -0.234 | 0.012 | 0.013 | 0.001 |
| **S7** | 0.453 | 0.013 | 0.016 | 0.003 | **S7** | 0.457 | 0.006 | 0.013 | 0.007 | **S7** | 0.454 | 0.009 | 0.012 | 0.003 |
| **O8** | -0.379 | 0.042 | 0.047 | 0.005 | **O8** | -0.375 | 0.027 | 0.032 | 0.005 | **O8** | -0.374 | 0.038 | 0.033 | -0.005 |
| **O9** | -0.380 | 0.041 | 0.048 | 0.007 | **O9** | -0.376 | 0.033 | 0.029 | -0.004 | **O9** | -0.374 | 0.027 | 0.041 | 0.014 |
| **N10** | -0.683 | 0.012 | 0.004 | -0.008 | **N10** | -0.680 | 0.007 | 0.002 | -0.005 | **N10** | -0.681 | 0.010 | 0.001 | -0.009 |
| **C11** | 0.223 | 0.100 | 0.108 | 0.008 | **C11** | 0.178 | 0.061 | 0.052 | -0.009 | **C11** | 0.246 | 0.068 | 0.072 | 0.004 |
| **C12** | -0.778 | -0.023 | -0.022 | 0.001 | **C12** | -0.830 | -0.021 | -0.014 | 0.007 | **C12** | -0.810 | -0.014 | -0.020 | -0.006 |
| **N13** | -0.428 | 0.055 | 0.042 | -0.013 | **N13** | -0.267 | 0.020 | 0.024 | 0.004 | **N13** | -0.397 | 0.043 | 0.011 | -0.032 |
| **N14** | -0.419 | -0.009 | -0.015 | -0.006 | **N14** | -0.410 | 0.010 | 0.000 | -0.010 | **N14** | -0.424 | 0.000 | -0.003 | -0.003 |
| **C15** | 0.488 | 0.020 | 0.023 | 0.003 | **C15** | 0.426 | 0.068 | 0.072 | 0.004 | **C15** | 0.423 | 0.045 | 0.049 | 0.004 |
| **C16** | -0.756 | -0.011 | -0.010 | 0.001 | **C16** | 0.136 | -0.004 | -0.006 | -0.002 | **C16** | 0.125 | -0.001 | 0.008 | 0.009 |
| **O17** | -0.438 | 0.043 | 0.042 | -0.001 | **C17** | -0.296 | 0.026 | 0.027 | 0.001 | **C17** | -0.283 | 0.012 | 0.021 | 0.009 |
|  |  |  |  |  | **C18** | -0.187 | 0.007 | 0.007 | 0.000 | **C18** | -0.109 | 0.018 | 0.018 | 0.000 |
|  |  |  |  |  | **C19** | -0.219 | 0.040 | 0.042 | 0.002 | **N19** | -0.231 | 0.045 | 0.050 | 0.005 |
|  |  |  |  |  | **C20** | -0.182 | 0.007 | 0.005 | -0.002 | **C20** | -0.102 | 0.014 | 0.016 | 0.002 |
|  |  |  |  |  | **C21** | -0.267 | 0.033 | 0.032 | -0.001 | **C21** | -0.255 | 0.024 | 0.026 | 0.002 |
|  |  |  |  |  | **O22** | -0.595 | 0.053 | 0.048 | -0.005 | **O22** | -0.475 | 0.058 | 0.063 | 0.005 |

[1] A. Elsamman, K.F. Khaled, S.A. Halim, N.S. Abdelshafi,Development of QSAR based GFA predictive model for the effective design of a new bispyrazole derivative corrosion inhibitor, J. Mol. Struct 1293 (2023) 136230, <https://doi.org/10.1016/j.molstruc.2023.136230>.

[2] A. Elsamman, K.F. Khaled, S.A. Halim, N.S. Abdelshafi,A critical view of the QSAR model for the prediction of a new bispyrazole derivative BPYR-P as a corrosion inhibitor for 304 SS in a 1.0 M HCl solution, J. Mol. Struct 1297 (2024) 136728, <https://doi.org/10.1016/j.molstruc.2023.136728>.

[3] K. Khaled, N. Abdel-Shafi,Int. J. Electrochem. Sci 6 (2011) 4077,

[4] Accelrys, Materials Studio Manual. Accelrys, USA, 2013.

[5] Anonymous, Health & Beauty Close - Up. 2011.

[6] B. Delley,From molecules to solids with the DMol^3^ approach, J. Chem. Phys 113 (2000) 7756,

[7] J.H. Friedman, Multivariate Adaptive Regression Splines, in: Technical Report no. 102. Stanford University , Laboratory for Computational Statistics, , 1988.

[8] A. Elsamman, K.F. Khaled, S.A. Halim, N.S. Abdelshafi,Development of QSAR based GFA predictive model for the effective design of a new bispyrazole derivative corrosion inhibitor, J. Mol. Struct (2023) 136230, <https://doi.org/10.1016/j.molstruc.2023.136230>.

[9] A.A. Farag, S.M. Al-Shomar, N.S. Abdelshafi,Eco-friendly modified chitosan as corrosion inhibitor for carbon steel in acidic medium: Experimental and in-depth theoretical approaches, Int. J. Biol. Macromol. 279 (2024) 135408, <https://doi.org/10.1016/j.ijbiomac.2024.135408>.
